# Past, Present and Future Perspectives on Halloysite Clay Minerals

**DOI:** 10.3390/molecules25204863

**Published:** 2020-10-21

**Authors:** Marina Massaro, Renato Noto, Serena Riela

**Affiliations:** Department of Biological, Chemical and Pharmaceutical Sciences and Technologies (STEBICEF), University of Palermo Viale delle Scienze, Ed. 17, 90128 Palermo, Italy; renato.noto@unipa.it

**Keywords:** halloysite nanotubes, historical background, chemical modification, supramolecular functionalization

## Abstract

Halloysite nanotubes (HNTs), clay minerals belonging to the kaolin groups, are emerging nanomaterials which have attracted the attention of the scientific community due to their interesting features, such as low-cost, availability and biocompatibility. In addition, their large surface area and tubular structure have led to HNTs’ application in different industrial purposes. This review reports a comprehensive overview of the historical background of HNT utilization in the last 20 years. In particular it will focus on the functionalization of the surfaces, both supramolecular and covalent, following applications in several fields, including biomedicine, environmental science and catalysis.

## 1. Introduction

Halloysite nanotubes (HNTs) are clay minerals belonging to the kaolin group, which due to their interesting properties have been widely investigated in recent decades. HNTs are dioctahedral 1:1 clay minerals present in soils, discovered for the first time by Juan Baptiste Julien d’Omalius d’Halloy in Belgium, after whom they were named by Pierre Berthier in 1826. The mineral can be found worldwide, in particular in wet tropical and subtropical regions and weathered rocks. Countries such as China, France, Belgium and New Zealand are rich in this clay.

It presents a chemical formula of Al_2_Si_2_O_5_(OH)_4_·*n*H_2_O, and according to its hydration state they can be classified as hydrated HNTs (Al_2_Si_2_O_5_(OH)_4_·2H_2_O) with an interlayer d_001_ spacing of 10 Å, and dehydrated HNTs (Al_2_Si_2_O_5_(OH)_4_) with 7 Å d_001_ spacing. The occurrence of water molecules in the interlayer space is believed to be the reason for the different halloysite morphologies which constitute the main difference with platy kaolinite. Depending on the extraction site, geological occurrence and crystallization conditions, halloysite can be found as tubular, spheroidal or platy-like particles.

**Figure 1:**
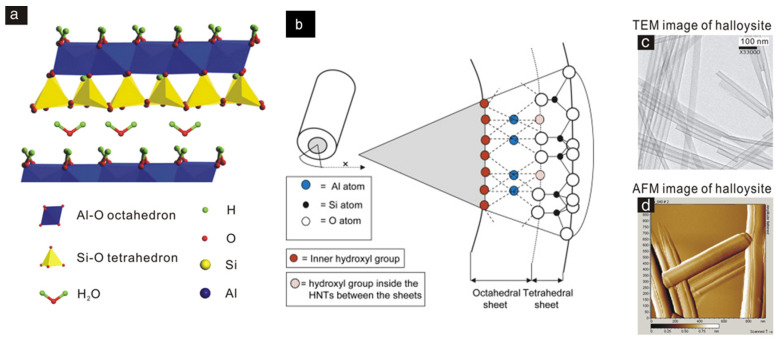
(**a**,**b**) HNTs crystal morphology and atomic structure; (**c**,**d**) Trasmission electron microscopy (TEM) and (Atomic force microscopy) AFM images of HNTs. Adapted with permission from [1,2].

Among these different morphologies, the tubular one is the most representative. Therefore, typical HNTs possess a hollow tubular structure in the sub-nanometer range with an aspect ratio of ca. 20. Structurally, HNTs are constituted by an external surface composed of siloxane (Si–O–Si) groups, an internal surface, a lumen that consists of a gibbsite-like array of aluminol (Al–OH) groups, and the presence of some Al–OH and Si–OH groups at the edges of the material or as structural defects (Figure 1). HNT walls consist of 10–15 bilayers, with a spacing of approximately 0.72 nm and have a density of 2.53 g/cm^3^ [1,2].

Thanks to their different chemical composition, the tubes undergo ionization in aqueous media in an opposite way, generating tubes with oppositely charged inner and outer surfaces [3]. This charge separation occurs in water within a wide pH range from 3 to 8 [4]. Experimentally, the charge separation is predicted by comparing the negative and positive values for the electrical ζ—potential of silica and alumina surfaces in water, respectively. The tubes have a length in the range of 0.2–1.5 μm, while the inner and outer diameters of the tubes are in the ranges of 10–30 nm and 40–70 nm, respectively [5,6,7]. Physico-chemical properties of HNTs are summarized in Table 1.

Currently, HNTs price ranges from US $600 per ton for application in fracking, to US $3000 per ton for fine chemical applications. It is easy to imagine that in the near future HNTs could replace the much more expensive carbon nanotubes ($500 per kg) and in many cases HNTs could be used in high technological applications where carbon nanotubes are not suitable.

Furthermore, HNTs are available at the scale of thousands of tons, compared to gram-scale for CNTs, and conversely to the latter they are biocompatible materials [9]. Compared with imogolite nanotubes, which are also naturally occurring, the pore size of HNTs is much larger; whereas conversely to boron nitride, which is chemically inert, HNTs can be modified on their surfaces by the grafting of suitable groups [10].

**Figure 2:**
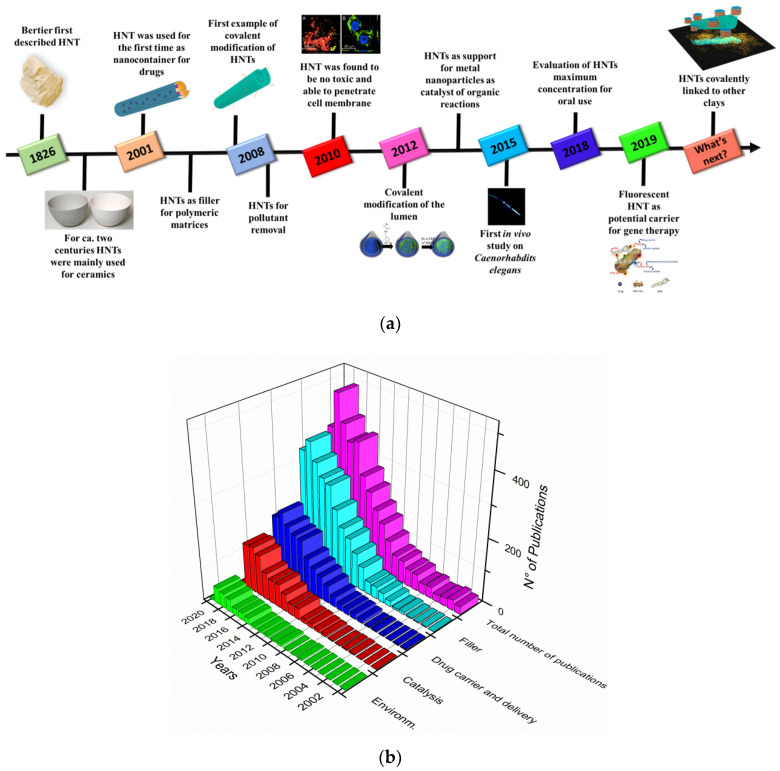
(**a**) A brief timeline for the development of HNTs; (**b**) Number of publications on HNTs sorted by year and for application. Data were collected from “Scopus”. The word “halloysite” is keyed into the “topic” search box (date of search: 22 September 2020). Adapted with permission from [11,12,13,14,15].

The development and broad application of HNTs based nanomaterials have rapidly advanced in recent years as indicated by the large number of publications from their advent as cheap and wide available nanocontainers in 2001 to the present day. This trend clearly reveals the global significance of HNTs and the intense interest of scientific research in this field (Figure 2).

Until 2001 the research on halloysite clay was only focused on the geological and mineralogical aspects of these clay minerals and their main use was in ceramic manufacturing.

Then, Price et al. proposed the use of HNTs as a low-cost alternative to more traditional microencapsulation systems for the delivery of hydrophobic compounds [16]. Their idea was the result of previous studies on lipid tubules formed from polymerizable phospholipids, used as nanocontainers for several hydrophilic and hydrophobic active agents such as oxytetracycline chloro-hydrate, proteins, and growth factors [17]. The authors thought to use inorganic HNTs for the encapsulation and subsequent release of tetracycline chloro-hydrate, khellin and nicotinamide adenine dinucleotide. Although this study advanced the use of HNTs as drug carrier system, it showed some limitations. At that time, the major drawback was the fact that the clay, being a natural material, varied from deposit-to-deposit and even within deposits, which at first attempt restricted applications to those where variations in entrapment rates and release characteristics are not critical.

However, the pioneering study of Price et al. laid the foundation for subsequent extensive studies of HNT clay mineral from a physico-chemical point of view.

The following year, Levis and Deasy reported on a detailed physico-chemical characterization of HNTs mined in New Zealand, which are relevant to subsequent investigations into their use for the production of novel delivery systems for drugs and other agents [18]. They found that by sieving HNT samples from New Zealand using 125 μm sieves, it was possible to obtain particles with an average median particle size of 27.9 μm. In addition, HNTs dehydrated state was not readily reversible and, therefore, the intercalated space was unlikely to be available for drug loading.

Zeta potential measurements confirmed the occurrence of a negatively charged external surface and a positively charged lumen, thus it was possible to load cationic drugs on the external surface to delay drug release. By exploiting this aspect, in the same year Lvov et al. synthetized by a self-assembly process ordered multilayers containing from 2 to 20 layers of tubes, glued together with polycation interlayers constituted of poly(ethyleneimine) [19].

Developing their studies, Levis and Deasy, in 2003, published the first complete report dealing with the use of halloysite for sustained delivery of drugs. They chose diltiazem chloro-hydrate and propranolol chloro-hydrate as drug models [20]. They found that the water-soluble cationic diltiazem chloro-hydrate bound to the polyanionic surfaces of HNTs, and showed a slightly sustained release effect on dissolution testing due to reversible chemisorption and/or hindered release from the drug loaded lumen. On the contrary, the use of a less water-soluble cationic drug, propranolol chloro-hydrate, allowed the authors to achieve a greater sustained release effect. In order to further delay drug release, they tried to load diltiazem chloro-hydrate from a polyvinylpyrrolidone solution into the halloysite, but the results were unsatisfactory.

Better results were obtained by coating the halloysite external surface with different cationic polymers, such as chitosan cross-linked with glutaraldehyde and polyethyleneimine (PEI). The last approach was also useful for the sustained release of tetracycline from halloysite for the treatment of periodontitis [21].

In another work, Lvov et al. filled the HNTs’ lumen with urease to obtain a biomineralization nanoreactor for carrying out enzyme-catalyzed inorganic synthesis [22]. The authors investigated the urease-catalyzed hydrolysis of urea in the presence of CaCl_2_. The formation of a CaCO_3_ precipitate inside the halloysite particles started immediately after urea decomposition into ammonia and CO_3_^2−^ ions, catalyzed by the entrapped urease. Urea decomposition and CO_3_^2−^ ion formation occurred in halloysite lumen whereas calcium cations diffused from the surrounding solution. The experimental findings showed that CaCO_3_ formation did not occur on the outer surface of the halloysite nanotube nor in solution, confirming the occurrence of the reaction inside the tubes.

Afterwards, scientific research on halloysite was mainly focused on HNTs’ application as filler to reinforce polymeric matrices.

Since the discovery of the beneficial effects of the addition of clay nanoparticles to polymers in 1965, several examples have been reported. The use of HNTs as additive started in 2006, when Guo et al. [23] found that, by adding HNTs to a polypropylene matrix, they obtained a nanocomposite which presented a remarkable enhanced thermal stability and a reduction in flammability with respect to the neat polymer. These findings were explained by the presence of HNTs, which can entrap the degradation products of polymer into the lumen resulting in effective delay in mass transport and a remarkable increase in thermal stability.

Over subsequent years, HNTs were mainly use as fillers for polymeric matrices. Several polymers have been considered and, in each case investigated, the addition of halloysite to the polymer matrices showed several advantages in the improvement of the physico-chemical properties of nanocomposites (Table 2).

However, despite the outstanding improvement of the physico-chemical performances of HNT/polymer nanocomposites, the poor interfacial interactions between the polymers and the clay nanoparticles, and the agglomeration of HNTs in the polymer matrix, limited their effectiveness as filler.

The functionalization of clay nanoparticles, both supramolecular and covalent, allowed these problems to be overcome.

The aim of this review is to deliver a comprehensive overview of the relevant advances in the field of halloysite nanotubes since the beginning of these extensive studies on their functionalization, both supramolecular and covalent, on both HNT surfaces, following developments in the field that year by year have led to the obtaining of innovative and smart nanomaterials which find application in several fields, including biomedicine, environmental science and catalysis.

The first section of the review will be focused on the supramolecular functionalization of both inner and outer surfaces of HNTs, the second will mainly address the covalent modification of the external surface and its advantages; finally, a short paragraph will be devoted to the covalent modification of the lumen and of interlayer spaces. For each modification investigated an overview of the proposed applications will also be considered. In addition, the chemical manipulation of clays used to improve properties will be briefly reviewed.

Hereinafter we refer to functionalized HNTs as those interacting by supramolecular forces with “guest” molecules; and to modified HNTs as those which react covalently with organo-silanes.

## 2. Supramolecular Functionalization

In the beginning, the researchers focused their attention on the supramolecular binding of several molecules by exploiting the different HNT charged surfaces. As stated above, HNTs possess a positively charged inner surface, which can interact with negatively charged or electron-rich molecules, while the external surface, being negatively charged, can successfully bind with positively charged molecules. In addition, both surfaces can also experience hydrogen bond interactions.

### 2.1. Functionalized Halloysite as Filler

The first examples of HNTs used as filler to reinforce polymeric matrices envisaged the utilization of the nanomaterial as it was. Progressing with these studies, the researchers realized that pristine HNTs showed some limitations. For example, HNTs were hardly an effective filler for rubber because of the unsatisfied interfacial bonding and agglomeration in the rubber matrix. In 2008, Guo et al., for the first time, exploited HNT surface modification to develop a novel nanofiller with improved interfacial properties. They modified the HNTs’ outer surface via electron transferring interaction, by the introduction of the electrons’ donor 2,5-bis(2-benzoxazolyl) thiophene (BBT). The HNTs/BBT hybrid was used as filler for polypropylene (PP) obtaining a nanocomposite with enhanced tensile and flexural properties in comparison with neat PP and PP/HNTs nanocomposite, due to better crystallinity [32].

The same authors also investigated the functionalization of HNTs with methyl-methacrylate (MMA) to obtain a stable dispersion of HNTs into styrene-butadiene rubber (SBR). A year later, to overcome the problems arising from the corrosive nature of MMA, they investigated the possibility of using sorbic acid (SA) to improve the performances of HNT/SBR nanocomposite [33]. The authors found that by adding sorbic acid to HNT/SBR, HNTs’ fillers were better dispersed in the polymeric matrices by the formation of hydrogen bonds between SA and HNTs. At the same time, SA was grafted onto the SBR backbone via radical copolymerization, increasing the interfacial bonding between SBR and HNTs via SA intermediated linkage.

Since then, several efforts have been made by the scientific community to find the best conditions to improve HNTs’ dispersion in different polymers [34].

In 2011, HNTs were functionalized with two different ionic liquids (ILs), namely, 1-methylimidazolium- and bis (1-methylimidazolium) mercapto-succinate [35], obtaining better dispersion into SBR compared to other functionalization methods. The ILs were bound on the HNTs’ outer surface by hydrogen bonds and then the HNT/ILs hybrid were reacted upon by means of a thiolene reaction with SBR. The obtained nanocomposite showed better performances in comparison with the SBR/HNT nanocomposite and these findings were successfully correlated with a more stable clay dispersion in the polymeric matrix.

**Figure 3:**
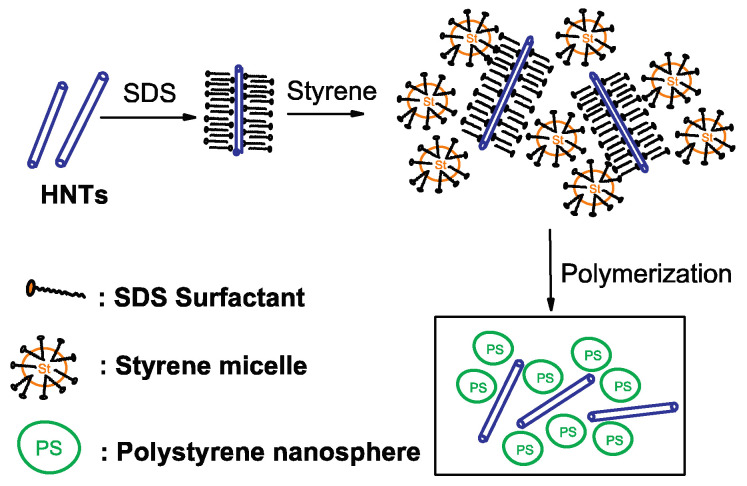
Schematic representation of the in situ polymerization of styrene in the presence of HNTs. Reproduced with permission from [36].

During these same years, very stable dispersion of HNTs in polymeric matrices was also obtained by the HNTs’ functionalization with surfactants. As example, Chan et al. [36] reported the preparation of high-impact polystyrene nanocomposites filled with individually dispersed HNTs via the emulsion polymerization of styrene, with HNTs functionalized with sodium dodecyl sulfate used as the emulsifier (Figure 3).

Fluorescent carbon nanodots (CDs) with antioxidant properties, obtained by the microwave mediated pyrolysis of citric acid in the presence of 1,2-ethylenediamine, were successfully loaded into HNTs’ lumen [37]. The complex obtained was used as filler for a rubber matrix where the CDs’ loaded halloysite ensured long-lasting radical-scavenging activity suppressing the oxidative process of the organic matrix. Furthermore, it was found that the rubber was preserved, retaining its physical and chemical properties after severe thermo-aging for 20 days at 100 °C which corresponds to many months of protection at lower service temperatures.

**Figure 4:**
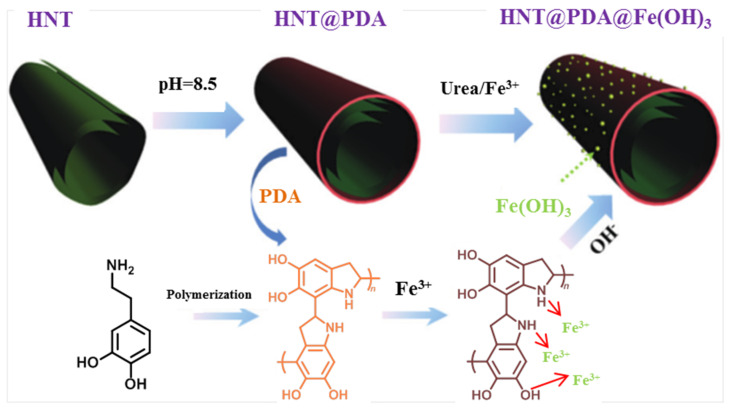
Preparation process of HNT@PDA@Fe(OH)_3_ nanohybrid. Reproduced with permission from [38].

In 2017, Wang et al. sequentially functionalized HNTs’ external surface with a biomimetic polydopamine (PDA) nanocoating and ultrafine Fe(OH)_3_ nanoparticles to prepare hierarchical HNTs@PDA@Fe(OH)_3_, with the aim of endowing epoxy resin (EP) with improved fire retardancy, thermal stability and mechanical properties (Figure 4) [38]. For instance, dynamic mechanical analysis (DMA) results revealed that the incorporation of HNTs@PDA@Fe(OH)_3_ into EP increased the storage modulus and glass transition temperature compared to pristine polymer.

The examples above reported shows that the functionalization of clay surfaces with different molecules allows fillers to be obtained which can improve the performance of polymeric matrices.

Simultaneously to these studies, researchers investigated the possibility of using HNTs as nanocontainers for molecules for application in biomedicine, catalysis and in the environmental field. The differently charged surfaces can be exploited for the immobilization of drugs, or biologically active molecules and pollutants.

### 2.2. Functionalized Halloysite for Environmental Purposes

In the first decade of the 21st century, several materials were used as adsorbents for pollutant removal such as activated carbon [39], silica [40], carbon nanotubes [41], etc., but their high operating costs limited their effective utilization. In 2007 the possibility was explored of using natural clay minerals as sorbent for environmental purposes. The advantages were soon clear. Indeed, clay minerals are cheaper than activated carbons and they also provide a highly specific surface area. In this context, halloysite gained a lot of attention for its peculiar physico-chemical properties and, more importantly, they were much cheaper and more easily available compared to the more well-known, carbon nanotubes.

The first example reported of the use of HNTs for pollutant removal dates back to 2008, when Liu and Zhao [42] studied the adsorption capacity of HNTs toward methylene blue, chosen as cation dye model. They found that HNTs could efficiently adsorb the dye showing a maximum adsorption capacity of 84.32 mg·g^−1^. Therefore, HNTs could be valuable candidates for large scale applications.

Two years later, to avoid rapid agglomeration and precipitation of HNT nanoparticles which often occurs, Bing et al. [43] functionalized halloysite with a surfactant, hexadecyltrimethylammonium bromide, and they used the new material as adsorbent for Cr(IV) removal.

A step forward towards the use of HNTs as adsorbent for pollutant was proposed in 2011 by Ma et al. [44]. They exploited the possibility of anchoring, by physisorption on HNT surfaces, magnetic nanoparticles. With this idea in mind, the authors synthetized Fe_3_O_4_ nanoparticles mainly on HNTs’ external surface obtaining a nanomaterial HNTs–Fe_3_O_4_ with an HNTs/Fe_3_O_4_ ratio of 1:1. Afterwards, they investigated the feasibility of the nanomaterial as sorbent for two cationic dyes, methylene blue and Nile red, and for methyl orange, chosen as anionic dye. The cationic dyes were successfully removed from aqueous solution as a consequence of favorable electrostatic interactions with the negatively charged HNTs; on the contrary, methyl orange was poorly adsorbed into the system. Thanks to the presence of magnetic nanoparticles, the nanomaterial was simply recycled by magnetic separation.

A year later, Zhang et al. used a similar nanomaterial for the removal of methyl violet [45].

Following a similar approach, TiO_2_ nanoparticles were deposited onto HNTs’ external surface by Chen et al., by means of a one-step solvothermal method [46]. The authors employed the prepared nanomaterial for the photodegradation of methanol and acetic acid. The systems showed pH sensibility and high photocatalytic activity in the degradation of methanol, and high photocatalytic activity in the degradation of acetic acid, due to the few radical needs consumed during the degradation reaction.

The in situ polymerization of pyrrole in HNTs; dispersion was carried out by Maity et al. to obtain poly-pyrrole-coated HNTs which were successful applied as adsorbent for Cr(VI) removal [47]. The so-obtained nanocomposite showed a promising adsorption capacity of 149.25 mg·g^−1^ at pH 2.0 at 25 °C and it was recyclable for at least three cycles without loss of its original removal efficiency. The most important aspect of this work was due to the fact that the authors tested the adsorption capacity of the nanocomposite on Cr(VI) contaminated groundwater and chrome mine wastewater and the results highlighted the feasibility of the material used as sorbent for the remediation of Cr(VI).

The introduction of per-fluorinated compounds in the inner lumen is another strategy to obtain advanced materials for several applications [48]. The modified HNTs with perfluoro-alkylated anionic surfactants formed kinetically stable aqueous dispersions due to the enhanced electrostatic repulsions exercised between the particles. This hybrid can be used for non-foaming oxygen nanocontainers in aqueous media. The gas release from supersaturated dispersions can be controlled by external stimuli and system composition. Halloysite was also functionalized with dioctyl sulfosuccinate sodium salt and the obtained nanomaterials were successfully employed as stabilizing oil-in-water emulsions [49].

In 2015, emerging nanomaterials for CO_2_ capture were obtained by impregnation of HNTs with poly-ethenimine [50]. A polymer loading of 50% allows a nanocomposite that showed a CO_2_ adsorption capacity of 2.75 mmol·g^−1^. Furthermore, this material showed quick kinetics and better stability in 10 cycles of CO_2_ adsorption/desorption behavior.

In the same year, Liu et al. [51] proposed electro-spun carbon doped TiO_2_/HNTs nanofibers as photocatalyst for the degradation of organic dyes. The experimental findings highlighted that the visible light photocatalytic efficiency of the nanofiber was enhanced with a moderate HNT doping amount of 8%, far greater than that of commercial anatase TiO_2_.

In 2016, Lazzara et al. proposed the double functionalization of both HNT surfaces by supramolecular interactions with cucurbit[8]uril molecules [52]. The obtained materials were used as nano-sponges for the capture of aromatic compounds. Due to the high hydrophobicity of the system, it was possible to remove pyrene from aqueous systems and toluene molecules from both liquid and gas phases.

In recent years, HNTs were used as filler for polydopamine, developing a novel nanocomposite with enhanced thermal stability [53]. The introduction of HNTs into the PDA matrix was performed in two different oxidation conditions exploiting the different polymerization ability of dopamine in alkaline or acidic media. Depending on the medium used, two different morphologies were observed in the final nanocomposite, according to the interaction established between HNTs and PDA. The feasibility of the nanocomposite as membrane coating for environmental purposes was evaluated by studying its adsorption capacity towards Rhodamine B, chosen as dye model.

In 2019, W_18_O_49_ nanocrystals were supported on HNTs by a hydrothermal process coupled with a calcination treatment to obtain a halloysite@W_18_O_49_ nanocomposite which showed a smaller bandgap, stronger light absorption, and higher photocatalytic activity than pristine W_18_O_49_ nanocrystals [54]. The photocatalytic activity was tested by studying the degradation of methyl orange. Good catalytic performances were explained both by the generation of abundant ·OH that participates in MO degradation from the conspicuous light adsorption of the W_18_O_49_ nanocrystals, and by the adsorption of MO onto HNTs, increasing, thus, its local concentration.

Recently, ZnO nanoparticles (ZnONPs) were immobilized onto HNTs’ surface by a one-pot synthesis (HNTs@ZnO) using, for the first time as Zn^2+^ precursor, the commercially available ZnONPs (possessing dimensions of ca. 100 nm) [55]. By adopting this procedure, supported ZnONPs with smaller dimensions (2.4 nm) than pristine ZnONPs were obtained. The characterization of the material showed that the HNTs@ZnO nanomaterial possessed an energy band gap value of ca. 2.65 eV, lower than that of pure ZnONPs (3.2 eV), highlighting a beneficial effect of HNTs on the photophysical properties of ZnO. The material developed was tested as photocatalyst for the degradation of organic dyes under visible light and as catalyst in the transesterification reaction of soybean oil to obtain biodiesel. In both reactions, the material showed good catalytic activity and recyclability.

Aguzzi et al. evaluated the effect of ZnONPs on HNTs for photoprotection application focusing attention on the radiation screening capabilities of the material [56]. The authors investigated different synthetic approaches to obtaining ZnONPs on HNTs and correlated the synthetic method with the adsorption capacity of the obtained materials. Their experimental findings highlighted that by adopting an adsorption method, small and quite uniform sized (10–30 nm) zinc oxide nanoparticles inside HNTs’ lumen were obtained. The UV-vis spectra of these nanocomposites revealed their ability to interact with a wide portion of UV-vis radiation which led to enhanced sun screening performances.

### 2.3. Functionalized Halloysite in Catalysis

From a catalytic point of view, HNTs possess several advantages, including high stability, resistance against organic solvents, and ease of disposal or reusability. In comparison to platy clays such as montmorillonite, kaolin and LAPONITE^®^ that are stacked in larger crystallites, halloysite does not need exfoliation and thus has a large surface area without any additional material treatment.

The first example of the use of HNTs as support for catalytic species dates back to 2008, when Nakagaki et al. proposed HNTs for the immobilization of metallo-porphyrins for hydrocarbon oxidation [57]. The results obtained showed the importance of HNTs as nanotubular support for catalytic purposes. Although this study reported the feasibility of HNTs as support, first attempts to immobilize metal nanoparticles onto HNTs failed.

For this reason, in 2009, Liu and Zhao reported the immobilization of silver nanoparticles with about 10 nm diameter onto mercapto-acetic acid functionalized HNTs, via the in situ reduction of AgNO_3_ by polyol process [58]. The Ag/HNTs catalysts were found to be active in the reduction of several aromatic nitro compounds to corresponding amino derivatives, and easily recyclable.

Some years later, following a similar idea, chitosan was assembled, by electrostatic interactions, on HNTs’ external surface and used for horseradish peroxidase (HRP) immobilization through cross-linking by glutaraldehyde [59]. By this approach, a maximum enzyme loading of 21.5 mg·g^−1^ was reached. Furthermore, the HRP retained its activity after 35 days of storage in contrast with neat HRP which retained only 27% of its original activity. Going further, chitosan coated HNTs were assembled by Xiang et al. in 2014 [60], to form nest-like porous microspheres which, after dopamine functionalization to achieve a biomimetic entity, were used for laccase immobilization. The large mesopore, hierarchical pore distribution, and poly(dopamine) modification led to a significantly enhanced capacity, as high as 311.2 mg·g^−1^ for laccase loading. Specific activity of 80% can be retained for the immobilized laccase. Furthermore, the microspheres showed excellent thermal and recycle-use stability.

The HNTs’ lumen was used by Zhang et al. to immobilize two typical industrial enzymes, namely α—amylase and urease, by a simple physical adsorption process [61]. The use of HNTs allowed the authors to overcome enzyme utilization limitations, such as inactivation with temperature. They found, indeed, that by heating the hybrid HNTs/enzymes for 60 min, both immobilized enzymes retained more than 80% activity. Furthermore, the enzymes retained more than 90% of their activity even after 15 days of storage. They also showed the recyclability of the system.

In 2015, the effect of pH on the immobilization of several enzymes (laccase, glucose oxidase, lipase, and pepsin) in the halloysite cavity was investigated by Lvov et al. [62]. Depending on the protein charge, different interaction sites on HNTs were observed. As example, negatively charged proteins present loading values of ca. 5–7 wt1% and their release from the halloysite lumen is extended over time. In addition, glucose oxidase entrapped into HNTs’ lumen showed an improved thermal stability as well as an extension of storage time. All adsorbed enzymes exhibited improved biocatalytic abilities depending on pH conditions.

Besides organic molecules, since 2012 several metals were stabilized by HNTs and the resulting nanomaterials were used as catalysts in different processes. One of the first examples reported in the literature, dealt with the immobilization of ruthenium nanoparticles by the wet impregnation method of RuCl_3_ onto HNTs, with a consecutive metal reduction at 450 °C [63]. One year later another approach was considered, which took into account the immobilization of preformed Ru NPs by polyol [64]. Both HNTs@Ru catalysts were applied to the preferential oxidation of CO in a H_2_-rich atmosphere (PROX). The performances of the two catalysts were evaluated as percentage of CO conversion and as CO_2_ selectivity. An improvement was found in catalytic activity by varying the synthetic method; in particular the second catalyst showed significantly higher CO conversion and CO_2_ selectivity than the counterpart obtained by traditional wet impregnation. Since the polyol reduction obtained small-sized and uniformly dispersed nanoparticles, this method produces a better catalyst for the PROX reaction.

Co_3_O_4_ nanoparticles were supported on HNTs by Li et al. [65] for application in the Fischer-Tropsch reaction. The cobalt nanoparticles were immobilized by two different methods, namely double-solvents and wet impregnation. Compared with the catalyst prepared by wet impregnation, the catalyst prepared by the double-solvent method prevented the Co_3_O_4_ particles from migration and agglomeration due to size-induced effects, thus showing higher CO conversion and C_5+_ selectivity for Fischer-Tropsch synthesis.

After their first study, in 2013 Nakagaki et al. improved the catalytic system they had developed and immobilized anionic iron(III) porphyrin on pre-calcinated halloysite nanotubes [66] to obtain a catalyst for oxidation of several substrates (namely cyclooctene, cyclohexane, or n-heptane) using iodosyl-benzene as oxidant agent. Good catalytic results and appreciable turnover numbers were achieved in comparison with the non-supported porphyrin.

The coating of HNTs’ external surface with polymers was also exploited by Kim et al. to attach palladium nanoparticles on poly(N-isopropylacrylamide)–halloysite nanocomposites, obtaining hydrogels which were successful applied as catalyst in the Suzuki reaction [67].

Graphitic carbon nitride (g-C_3_N_4_) was combined by Huo et al. with ZnONPs supported onto halloysite via a facile calcination method [68]. This was tested as photocatalyst for the degradation of tetracycline under visible light irradiation. It was found that the g-C_3_N_4_-ZnO/HNTs nanocomposites showed much higher photocatalytic activity than those of either individual ZnO or C_3_N_4_, and than ZnO/HNTs arising from the combination of the metal oxide with HNTs.

### 2.4. Functionalized Halloysite as Drug Carrier

Although the first studies on halloysite demonstrated its feasibility as drug carrier, in the following years only a few reports dealt with this topic. Their unique physico-chemical properties and the presence of a hollow cavity available for the encapsulation of biologically active species, made HNTs an interesting material for biological applications. On the other hand, research into HNTs was at its beginning and there was little information about halloysite safety.

In 2008, Viseras et al. loaded 5-amino salicylic acid (5-ASA) on HNTs to investigate the possibility of achieving the controlled and sustained release of the drug for the preparation of colon targeted release systems [69]. They found that the interaction between 5-ASA and HNTs was a combination of two separate processes. The first of these was the rapid adsorption of the drug at the external surface of the clay mineral particles, while the second was adsorption occurring inside the HNTs’ pores and aggregates and was therefore slower. This hypothesis was confirmed a year later by the investigation of the solid hybrid 5-ASA/HNTs [70]. It was found, by adopting different techniques, that there is an actual interaction between 5-ASA and the HNTs; and more importantly, HREM microscopy coupled with XEDS analysis of stained samples allowed one the location of the drug at the surface to be distinguished, but also inside of the lumen, according to the different mechanism found by adsorption studies.

Until 2010 the use of HNTs in the biomedical field was limited to the study of the interaction of drugs with its surfaces. In this year, Leporatti et al. reported an outstanding work in which they assessed halloysite toxicity and its uptake by the cells [11]. By studying the cellular uptake of fluorescently labeled HNTs by means of confocal laser scanning microscopy by two different cancer cell lines, namely HeLa and MCF-7, the authors found that HNTs can efficiently penetrate the cell membranes localizing in the perinuclear region surrounding the cell nuclei. Furthermore, MTT and Trypan blue tests highlighted the biocompatibility of the nanomaterial, demonstrating that it is not toxic up to concentrations of 75 mg·mL^−1^. This study is important in halloysite research since it focused the attention on the biocompatibility of halloysite and its internalization by cells, which represents the main prerequisites for safe usage of the nanomaterial in the delivery of biologically active substances.

In 2015 Shoaib et al. evaluated the in vitro cytotoxicity of HNTs against hepatocarcinoma cells HepG2 and colorectal carcinoma cells HCT116 to assess the oral use of the clay [71]. Moreover, HNTs were tested for their cytogenetic toxicity against human peripheral blood lymphocytes. The experimental findings confirmed that HNTs are safe materials and thus can be used in pharmaceutical formulations.

A step further towards the use of HNTs for biological applications was successively made when the first in vivo studies began. In this year, Fahkrullin et al. [14] reported the first example of the evaluation of halloysite toxicity in vivo on free living nematodes (worms), Caenorhabditis elegance. It was demonstrated that HNTs were safe materials within a wide range of concentrations since they did not provoke any damage to the organism.

Eventually, in 2018, Hu et al. [72] investigated, for the first time, the hepatic toxicity of purified halloysite in mice via the oral route. They found that the oral administration of HNTs was beneficial for mice growth at low dose (5 mg·kg^−1^ per body weight (BW)), but it became dangerous at middle or high dose in a dose-dependent manner (from 50 to 300 mg·kg^−1^ BW). Their experiments showed that HNTs’ toxicity could be related to an Al accumulation in the mouse liver after 30 days of prolonged administration. This study allowed the definition of the maximum concentration of HNTs admitted for an oral use, of ca. 20 mg·kg^−1^ BW.

Most recently, Rhozina et al. reported for the first time the absence of negative effects of HNTs on the spontaneous assembly of cells into multicellular mono- and mixed spheroids, showing that they could be used in the near future for applications in tissue engineering [73].

Once it had been assessed that halloysite is not toxic for the cells, since 2008 extensive research began into its use as cheap alternative to the common used drug carrier systems.

**Figure 5:**
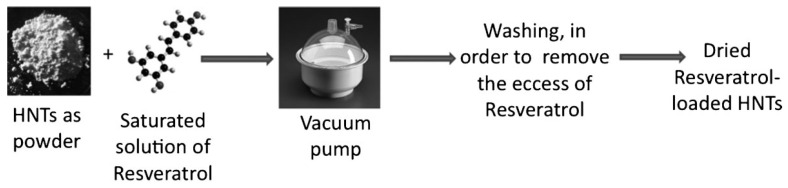
Scheme of the resveratrol loading inside HNTs lumen. Reproduced with permission from [74].

Resveratrol, a poor water soluble drug, was loaded onto HNTs from aqueous solvent containing ethanol, which provided higher solubility for the drug [74]. The loading was performed by a series of vacuum cycling of halloysite suspension in saturated solution containing resveratrol. In this approach, the air located inside the tubes was replaced by the drug solution (Figure 5). The authors found that the best experimental conditions to ensure the highest loading were achieved by using as solvent an aqueous solution containing 70% of ethanol and with a saturated solution of resveratrol with a concentration of 0.5 M. In this way, the entrapment efficiency was 99.7%. The also studied the resveratrol release in physiological conditions. The results showed a full release after 48 h with an initial burst effect where ca. 20% of the total drug loaded was released in the first 15 min. Finally, they evaluated the cytotoxicity of the system, by MTT test on MCF-7 cells showing promising cytotoxicity after 96 h of treatment.

In the same year, Lvov et al. exploited the use of halloysite as nanocontainer for gentamicin for antibiotic prolonged release [75]. They used the carrier system as filler for poly-(methyl)methacrylate (PMMA) to produce PMMA as bone cement. The introduction of halloysite provided a slow antibiotic release without compromising the composite mechanical strength. Antibacterial tests highlighted that the gentamicin release could inhibit the bacterial growth of Escherichia coli and Staphylococcus aureus. Up to now different chemically and biologically active molecules with a negative electron density have been successfully loaded on HNTs including insulin [76], vancomycin [77], bovine serum albumin [78], isoniazid [78], carvacrol [79], etc. [80,81,82].

Improvement in the biological activity of this kind of system can be obtained, for example, by further slowing the release of the active component. Based on these premises, in continuation of their work, the same authors reported an LbL assembly of polyelectrolyte multilayer shells on HNTs to retard the release of a drug loaded into HNTs’ lumen [83]. Their experimental findings showed that this approach decreased the release rate of dexamethasone, chosen as model, by four times in comparison with pristine nanotubes. Indeed, the drug was fully released from pristine HNTs within 7 h; on the contrary, a release in 30 h was achieved after coating the tubes with the polyelectrolytes.

A similar approach was chosen in 2012 by Garea et al. who coated HNTs with poly-(vinyl alcohol) to slow down the release rate of diphenhydramine hydrochloride from HNTs’ lumen [84].

In 2014, Jia et al. exploited an electrostatic layer-by-layer method to deposit a positively charged PEI on the HNTs external surface (f-HNTs) [85] for the delivery of siRNA into cancer cells and noninvasively imaged the process simultaneously. To reach this goal, they covalently conjugated the nanocomposite with siRNA, CdSe QDs, to obtain fluorescent label probes. The experimental findings demonstrated that the so-obtained f-HNTs carriers exhibited the efficient intracellular transporting and high delivery efficiency of siRNA. Furthermore, the f-HNTs-mediated siRNA improved the antitumor activity of siRNA which could effectively induce the knockdown of the target surviving gene in PANC-1 cells.

Zhang et al. reported the synthesis of superhydrophobic gated nanocontainer based on HNTs and poly-organo-silanes (POS) as molecular gate for sustained release of diclofenac sodium via the oral route [86]. The nanocontainer was synthetized by a two steps procedure: firstly, the diclofenac sodium was loaded onto HNTs reaching an adsorption capacity of 50 mg·g^−1^. The loading was performed by vacuum cycling and most of the drug molecules were entrapped into the positively charged lumen of HNTs via electrostatic interactions. Secondly, the co-condensation of hexa-decyltriethoxyl-silane (HDTES) and tetra-ethoxy-silane (TEOS) generated a layer of POS film on the surface of the HNTs/drug.

Coating halloysite with suitable end stoppers was another modification route adopted to slow down the release of active components from HNTs’ lumen. For example, by using benzotriazole-copper tube coating, Lvov et al. reached a prolonged release of brilliant green up to 200 h [87], longer than that achieved in the case of pristine halloysite (complete release after 3 h).

Two years later, the same authors [88] fabricated a novel drug delivery system based on HNTs loaded with brilliant green coated with dextrin to clog the tube opening. When the nanomaterial is taken up by the cells, the sugar can be cleavable by intercellular glycosyl hydrolases releasing the drug loaded into the lumen.

In 2014, the functionalization of HNTs’ inner surface with suitable organic molecules, allowed Lazzara et al. to develop nanocomposites where different tubes were linked to form microfibers. This was possible by the design of ad hoc molecules with azide and alkyne terminal groups which could be linked together by a click reaction. These microfibers were dispersed in chitosan and hydroxypropyl cellulose matrices [89] observing an increase of physico-chemical performances when compared to the nanocomposites obtained in the presence of pristine HNTs. These nanocomposites proved promising for application in food packaging. In this context, a year later, Gorrasi et al. filled a pectin matrix with rosemary essential oil loaded into HNTs. In order to avoid evaporation of essential oil and to increase the loading, in 2016 a novel filler was developed by functionalization of both HNT surfaces with cucurbit[6]uril (CB[6]) units for the loading of peppermint essential oil [90]. The HNTs/CB[6] systems were mixed by an optimized casting process into pectin, obtaining a nanocomposite with superior antioxidant and antibacterial activities.

Following the above proposed idea salicylic acid loaded HNTs were used as filler for pectin to fabricate biofilms with excellent thermo-mechanical properties as well as effective antimicrobial activity against several bacteria including Salmonella, Pseudomonas Aeruginosa, Escherichia Coli and Staphilococcus aureus [91]. In the same way, HNTs loaded with carvacrol and thymol were dispersed into low-density polyethylene for application in the food package field [92].

**Figure 6:**
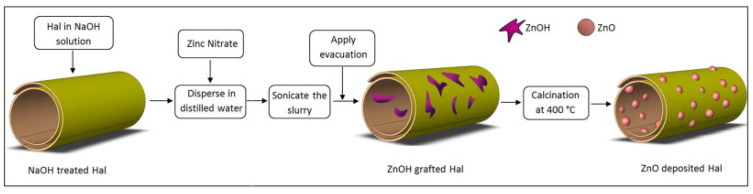
Schematic representation of the deposition process of ZnO nanoparticles (ZnONPs) onto HNTs. Reproduced with permission from [93].

In 2015, Pasbakhsh et al. proposed a ZnO deposited on HNTs’ filler to enhance the properties of poly(lactid acid) (PLA) films for food packaging (Figure 6) [93]. ZnO nanoparticles were deposited on the outer surface of HNTs and encapsulated inside their lumen using a novel, two-step solvothermal method. After their incorporation into the PLA matrix, it was found that the nanocomposite showed improved mechanical properties and exceptional antimicrobial activities against E. coli and S. aureus, compared to neat polymer.

The first pharmaceutical formulation based on HNTs appeared in the literature in 2017 when Lvov et al. [94] prepared targeted tablets containing a pharmaceutical excipient with excellent compression properties. The authors blended microcrystalline cellulose, colloidal silicon dioxide, magnesium stearate and croscarmellose sodium in the presence of the drug loaded HNTs, used as filler. Remarkably, release experiments showed that the drug can be retained in the tablet formulation for a long time (up to 20 h).

Sandri et al. reported a nanocomposite made of chitosan oligosaccharide/halloysite with a chitosan oligosaccharide/HNT weight ratio of 0.05 for application in wound healing [95]. The nanocomposite showed a good biocompatibility in vitro toward normal human dermal fibroblasts and by in vitro wound healing test an enhanced cell proliferation (cells in S-phase) was observed, rather than simple fibroblast migration. In vivo wound healing tests on murine model showed that after seven days of treatment, an early re-epithelialization process, an advanced degree of hemostasis and angiogenesis occurred.

**Figure 7:**
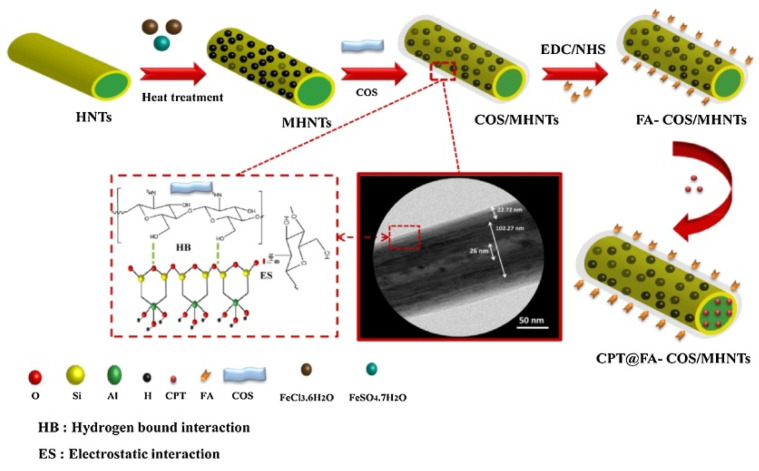
Schematic representation of the synthesis of camptothecin CPT@FA-COS/MHNTs. Reproduced with permission from [96].

Similarly, chitosan oligosaccharides were assembled on Fe_3_O_4_ functionalized halloysite by a simple solid-liquid interaction (COS@MHNTs) [96]. To enhance the therapeutic efficiency of the prepared nanocarrier, ligand conjugation and magnet targeting were combined. Folic acid (FA) was conjugated on the surface of COS@MHNTs via *N*-(3-dimethylaminopropyl)-*N*/-ethylcarbodiimide/*N*-hydroxysuccinimide coupling, yielding multi-targeted drug carrier based magnetic HNTs for the delivery of camptothecin (CPT) (Figure 7). The obtained delivery system exhibited high superparamagnetic properties and excellent receptor-specific targeting effects for Caco-2 cells and shows an outstanding usefulness in killing cancer cells. Concerning the drug loading, it showed an adsorption capacity of 227.10 mg·g^−1^ and a sustained release up to 60 h in acidic conditions.

Coating of HNTs with poly(sodium-p-styrene-sulfonate) (PSS) was found to be a good strategy to enhance the biocompatibility of the nanomaterial [97]. The subsequent loading into the lumen of a type-II photosensitizer indocyanine green (ICG) allowed the synthesis of a material for applications in phototherapy. The HNTs-PSS-ICG nanocarrier, without further tethering targeting groups, was shown to associate with the membrane of giant unilamellar vesicles (GUVs) via Pickering effects. Application of a HNTs-PSS-ICG nanocarrier to human breast cancer cells gave rise to a cell mortality as high as 95%. Coating again with MDA-MB-436 cell membranes endowed HNTs-PSS-ICG with a targeting therapy performance against breast cancer, which was confirmed by in vivo experiments using breast cancer tumors in mice. The membrane-coated and biocompatible nanocarrier preferentially concentrated in the tumor tissue, and efficiently decreased the tumor volume by a combination of photodynamic and photothermal effects upon near-infrared light exposure.

Recently, halloysite based hydrogels with a “turn-on” fluorescence character upon H_2_O_2_ was prepared and used to develop a H_2_O_2_-responsive drug delivery system [98]. In this work the authors prepared a PVA based hydrogel where a boronic acid modified fluorescein with a H_2_O_2_ sensible linkage was chemically introduced. After cleavage of this specific bond, fluorescein was released and the hydrogel became fluorescent. The introduction of a drug loaded HNTs into this hydrogel led to the formation of a nanocomposite that could be degraded in the presence of H_2_O_2_ at pathological concentration, in which the “initial burst effect” was suppressed. Moreover, a good linear relationship was achieved between the release rate and fluorescence intensity.

Nanocomposite films based on aldehyde-modified carrageenan, gelatin and halloysite nanotubes (AD-Carr/Gel/HNTs) were obtained by a solution casting process [99]. This novel nanocomposite showed no hemolysis and no cytotoxicity towards NIH3T3 fibroblast cells and therefore it should be promising for application in the tissue engineering field.

Most recently, halloysite nanotubes were used to produce cell-recognizing silica imprints capable of the selective detection of human cells [100]. The authors of this work used HeLa cells to template silica inorganic shells doped with HNTs. After sonication, the shells were destroyed forming polydisperse hybrid imprints that were used to recognize HeLa cells in liquid media supplemented with yeast. The methodology reported moved forward the use of HNTs in biomedical and clinical research, since it could be used for designing new methods for the selective recognition of normal and tumor cells.

## 3. Covalent Modification of the External Halloysite Surface

The example reported above showed how it was possible to manipulate HNTs with different functionalization, obtaining nanomaterials with tunable properties for various applications. However, sometimes pristine halloysite shows only weak interactions with guest molecules through hydrogen bonding or van der Waals forces and fast and non-controlled release. To avoid these drawbacks, it is possible to exploit a covalent modification on the HNTs’ surfaces, in particular the external one. The first example of covalent modification of HNTs’ surface dates back to 2008, when Kepert et al. reported the possibility of grafting organo-silanes via condensation, between hydrolyzed silanes and the surface hydroxyl groups of the HNTs located on the edges or on external surface defects [101].

The grafting reactions can occur in toluene, in water/alcohol mixtures or under solvent-free conditions under microwave irradiation. Since then, a variety of organo-silanes, bearing different terminal groups, were used for the HNT modification. Usually, the amount of silane grafted onto the HNTs’ surface has been estimated by thermogravimetric analysis (TGA) measurements. More recently, Licandro et al. reported a new method based on the use of Fmoc groups as probes covalently bound to 3-aminopropyltrimethoxysilane (APTES) and quantified by UV-vis after their release from the HNT–APTES–Fmoc system [102].

From the beginning, the most studied organo-silane for the modification of HNTs’ outer surface was APTES. First studies were focused on the development of valuable drug carrier systems and, year by year, different modification methods were adopted to overcome the problems often associated with drug administration. The use of organo-silane modified HNTs for industrial applications grew some years later, simultaneously to the assessment of HNTs as sorbent for pollutants or support for catalytic species.

The remaining sections will be focused on the different organo-silanes grafted onto HNTs’ external surface and the modifications adopted by researchers to obtain novel nanomaterials for application in several fields, including biomedicine, environmental science and catalysis.

### 3.1. Amino Modified Halloysite (3-Aminopropyltriethoxysilane (APTES) Grafting)

In 2013, Yuan et al. reported one of the first examples of amino functionalized halloysite for biological application [103]. They demonstrated that by grafting APTES onto HNT surface it was possible to achieve a higher loading of ibuprofen, chosen as drug model, in comparison to unmodified HNTs, because of the presence of favorable electrostatic attraction interaction between the positively charged amino groups on HNTs and the negatively charged carboxylic groups present in the ibuprofen molecule. A year later, the same authors reported that the advantage of modification was not only related to the increased loading, but it was also important to slow down the kinetic release of the drug, achieving a controlled and sustained release over time [104].

From then, different species were immobilized onto APTES modified HNTs for several purposes. It was demonstrated that HNTs-NH_2_ nanomaterial can load aspirin in a greater amount with respect to the pristine (11.98 wt % and 3.89 wt % for modified and unmodified HNT, respectively) [105]. The drug molecules were successively released in a controlled manner for ca. 16 h. APTES modified HNTs were used as carrier for the delivery of antisense oligodeoxynucleotides (ASODNs), as a therapeutic gene for targeting surviving [106].

An electrostatic self-assembly process was adopted in 2014 by Liu et al. to load HNTs on the surface of reduced graphene oxide sheets [107]. This approach was made possible by the modification of HNTs surface with APTES which led to a positively charged surface under acid conditions. The nanocomposite was easily separated in aqueous solution and it showed promising adsorption capacities towards Rhodamine B. Therefore, it could be useful for environmental purposes. In addition, it showed superior performance as electrode material in supercapacitors for energy storage issues.

According to the high affinity of amino groups towards metal ions, APTES modified HNT nanomaterials were successful applied for the immobilization of cations.

In 2015, Baglioni et al. reported, for the first time, the immobilization of Sr(II) on HNTs-NH_2_ and its dispersion into 3-hydroxybutyrate-co-3-hydroxyvalerate scaffold for application in the biomedical field [108]. Both polymer and metal were chosen since they have shown promising performance in bone tissue regeneration. In order to simulate the application of the nanocomposite as a coating onto synthetic bone grafts, it was prepared by spin-coating onto glass surfaces, whose chemical reactivity is similar to that of the ceramic materials commonly used in synthetic bone grafts. After exposure of the nanocomposite coated glass to para-physiological conditions, it was found that more than 60% of the initially loaded Sr(II) was retained after 28 days of degradation; thus, HNTs can retain a consistent amount of Sr(II) over time, suggesting that the nanocomposite bioactivity lasts long enough to allow the local regeneration of bone tissue.

APTES modified HNTs were used to fill polyvinylidene fluoride nanofiltration membranes to remove heavy metal ions from wastewater [109].

Subsequently, Kumar-Krishnan et al. loaded AgNPs onto HNTs-NH_2_ nanomaterial for the immobilization of the enzyme glucose oxidase (GOx) [110]. Thanks to the presence of amino groups on HNTs, the GOx immobilization was improved and revealed a high electrocatalytic activity for glucose reduction and the facilitation of enhanced charge transport.

In 2018, Pettignano et al. proposed the use of HNTs-NH_2_ as adsorbent for the removal of Pb^2+^ ions. They found that the adsorption ability of amino-functionalized HNTs-NH_2_ toward Pb^2+^ was considerably higher than that of p-HNTs in the same experimental conditions and that the obtained nanomaterial showed a noticeable reuse capability [111].

Starting from the amino groups present on the HNTs’ external surface, one can envisage the possibility of attaching polymers or small organic moieties on HNTs by amide condensation. In 2015 a new strategy was proposed to introduce stimuli-responsive polymers to tune the HNTs’ properties. One of the chosen polymers was a thermo-responsive one, specifically the poly-(*N*-isopropylacrylamide) (PNIPAAM) [112], which shows a low critical solution temperature (LCST) around 32 °C, after which the polymer brushes collapse on the HNTs’ surface. By exploiting this feature, the authors of the work constructed a carrier system to achieve a targeted release of a drug by changing temperature. They demonstrated that the loading of curcumin molecules on the HNTs-PNIPAAM system could be performed at 25 °C, whereas the drug molecule could be easily released at 37 °C. A year later, the same authors used the developed nano-system as support for PdNPS and its catalytic activity was tested in the Suzuki reaction between phenyl boronic acid and a series of aryl halide [113].

Following a similar procedure both poly(*N,N*-dimethyl-aminoethyl methacrylate) and poly(amidoamine) (PAMAM) were successful attached on HNTs-NH_2_ and the obtained systems were used for the loading and release of diphenhydramine hydrochloride, diclofenac sodium, chlorogenic acid, ibuprofen and salicylic acid [114,115].

**Figure 8:**
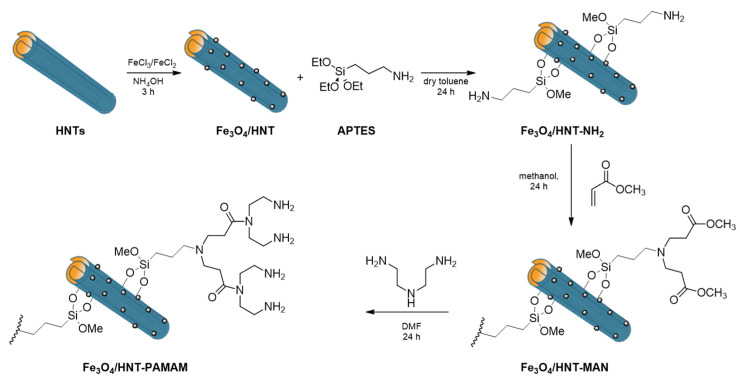
Scheme of the synthesis of poly(amidoamine) PAMAM modified Fe_3_O_4_@HNTs-NH_2_ nanomaterial.

Recently, PAMAM modified magnetic HNTs were successfully used for heparin recovery (Figure 8) [116]. Results showed a high efficiency of the system in the capture of heparin, both in terms of capacity and rate of adsorption, better than those obtained with Amberlite FPA98 Cl, a commercially accessible resin used in heparin extraction. The greater efficiency was explained by the presence of electrostatic attraction interactions between the PAMAM functional groups in the nanocomposites and the sulphate groups of heparins. Simultaneously, the same authors investigated the quaternization of the −NH_2_ groups of the APTES modified HNTs using methyl iodide to create permanent positive charges along the functionalized groups of the HNTs’ surface (HNT−NR_3_^+^), leading to a cationic polyelectrolyte character [117]. In this way, they optimized the recovery of heparin in alkaline conditions.

APTES modified HNTs were also used as a scaffold to graft polyethyleneimine (PEI) by Liu et al. [118] to develop vectors for the loading and intracellular delivery of DNA. In this work from 2017, in order to avoid cell injury and inflammation caused by long tubes, the authors firstly treated HNTs by ultrasound to shorten them, and then the grafting of PEI onto HNTs external surface (HNTs-PEI) occurred. The HNTs-PEI hybrids were used for delivery of pDNA. The biological assays on the hybrid complex showed that it possesses low toxicity and reasonable blood compatibility. In addition, the in vitro transfection results highlighted high transfection efficiency towards 293T and HeLa cells and, therefore, the HNTs-PEI/pDNA could be promising for application in gene therapy towards many diseases such as cancer.

Although the above reported system showed promising transfection efficiency toward tumor cells, some limitations are still associated with its use; first of all the cytotoxicity of the complex should limit the application in gene delivery. Therefore, a year after their study of HNTs-PEI/pDNA, the same authors proposed PAMAM modified HNTs (PAMAM-g-HNTs) to load siRNA and reduce the expression of the VEGF gene in breast cancer cells [119]. PAMAM-g-HNTs showed good cytocompatibility toward HUVECs (84.7%) and MCF-7 cells (82.3%) even at a concentration as high as 100 μg/mL. PAMAM-g-HNTs/siRNA exhibited enhanced cellular uptake efficiency of 94.3% compared with Lipofectamine 2000 (Lipo2000)/siRNA (83.6%). PAMAM-g-HNTs/small interfering RNA-vascular endothelial growth factor (siVEGF) led to 78.0% knockdown of cellular VEGF mRNA and induced 33.6% apoptosis in the MCF-7 cells, which is also much higher than that of Lipo2000/siVEGF. In vivo anticancer results demonstrated that PAMAM-g-HNTs/siVEGF treated 4T1-bearing mice showed enhanced anti-cancer efficacy than the Lipo2000/siVEGF group.

The amino functionalized halloysite was also used for laccase immobilization [120]. The functionalization improved the laccase loading and activity recovery with respect to pristine HNTs. In addition, the modified HNTs showed enhanced thermal and storage stabilities with excellent reusability.

**Figure 9:**
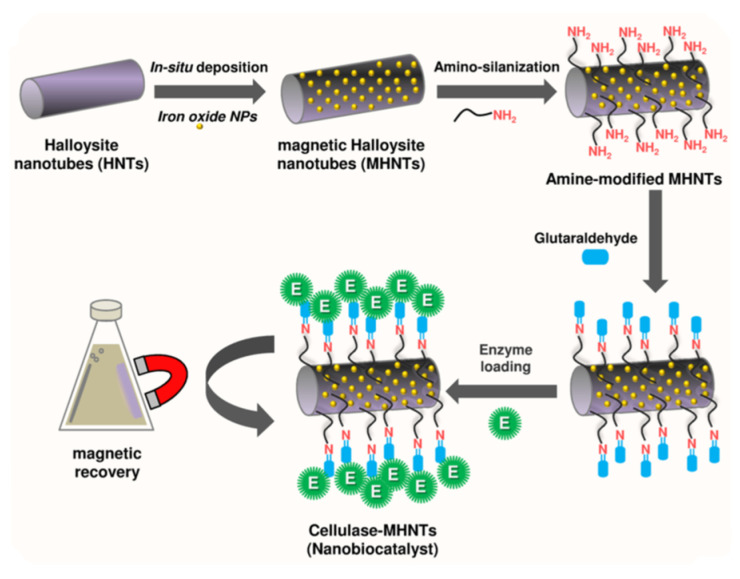
Schematic representation of the synthesis of cellulase-MHNTs (magnetic HNTs). Reproduced with permission from [121].

Similarly, in 2020 cellulase enzyme was grafted on magnetic HNTs’ (MHNTs’) external surface for biocatalytic purposes (Figure 9) [121]. It was demonstrated that the nanomaterial exhibited an excellent catalytic activity at elevated temperatures, ionic liquid-tolerant characteristics, hydrolyzation continuously over longer durations, and eco-friendly attributes, features which made the cellulase-MHNTs amenable for high cellulose conversion.

HNTs-NH_2_ were also used for the linkage of small antioxidant molecules onto HNTs’ external surface. For instance, in 2016, Amorati et al. linked trolox molecules on HNTs, developing a nano-antioxidant system, the activity of which could be restored if another antioxidant molecule was loaded inside HNTs’ lumen [122]. The authors demonstrated the latter aspect by performing an antioxidant experiment after loading of quercetin into the HNTs-Trolox system.

Based on the same experimental procedure, Fmoc-phenylalanine (FmocPhe) was linked to HNTs. The amino-acid is important from a biological point of view, since it could form stable hydrogels which can find application in tissue engineering and in the drug carrier fields. The so-obtained HNT nanomaterial was thus used as filler for FmocPhe hydrogels [123]. Due to the functionalization, interface properties of HNTs were improved and the filler was uniformly dispersed in the hydrogel matrix. This nanocomposite was used for the delivery of camptothecin for cancer treatment.

Last year, APTES modification of previously prepared Fe_3_O_4_@HNTs (MHNTs) nanomaterial was used as starting material for the linkage of cyclodextrin units, via EDC mediated amide condensation followed by folic acid (FA) conjugation via a PEG linker for the targeted isolation of cancer cells from whole blood samples [124]. The FA-loaded MHNT nanoparticles were able to capture different FR-overexpressing cancer cell lines with high efficiency, specificity and sensitivity, and exhibited high selectivity for cancer cells compared to the normal HEK 293 T cells.

**Figure 10:**
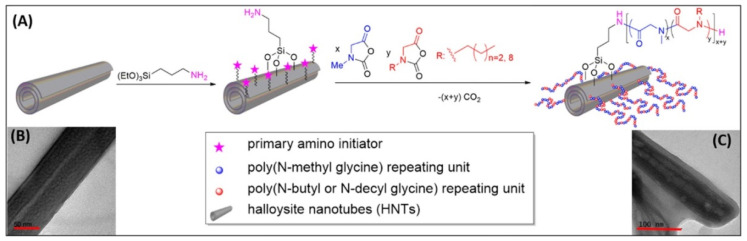
(**A**) Schematics showing the functionalization of HNTs with amphiphilic poly-peptoids by the surface-initiated polymerization method. (**B**) Representative TEM images of p-HNTs and (**C**) M80B20-g-HNTs in the dry state. Reproduced with permission from [125].

Amino modified HNTs were used as initiators for the ring-opening polymerization of *N*-substituted glycine-derived *N*-carboxy-anhydrides in varying monomer feed ratios to obtain poly-peptoids grafted on HNTs as emulsion stabilizers toward oil spill remediation (Figure 10) [125]. HNTs functionalized with poly-peptoids having appropriate hydrophilicity and lipophilicity balance (HLB) characteristics (HLB = 12.0–15.0) were found to be significantly more effective stabilizers for the oil/water emulsion relative to the pristine HNTs.

**Figure 11:**
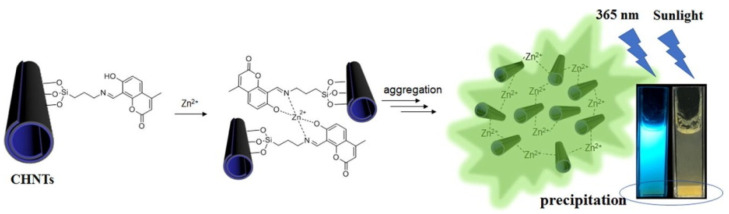
Synthesis of coumarin modified HNTs and their application as Zn^2+^ sensors. Reproduced with permission from [126].

More recently, coumarin derivative 7-hydroxy-2-ox-2*H*-chromene-5-carbaldehyde was covalently attached on amino functionalized HNTs via formation of a Schiff base [126]. This compound was chosen by the authors as fluorescence donator for detection and removal of Zn(II). The prepared coumarin modified HNTs showed, indeed, a selective turn-on fluorescence response in the presence of Zn^2+^ ions (Figure 11).

The same covalent linkage was adopted by Cauteruccio et al. to graft thialicene molecules (THA) onto HNTs [127]. Tetrathia[7]helicenes are very promising DNA intercalators, which need an appropriate carrier for biomedical applications to overcome their poor aqueous solubility. By anchoring the THA molecules on HNTs by the formation of a Schiff base, the authors achieved a triggered release of the active component in acidic environment, after the hydrolysis of the imine bonds.

Starting from APTES modification, Sohn et al. introduced carboxylic groups onto HNTs’ surface (HNTs-COOH) in order to improve the aqueous stability of HNT dispersion at different pH conditions [128]. The authors found that, by adjusting the solution pH, the aggregation/dispersion properties of HNTs-COOH could be controlled.

**Figure 12:**
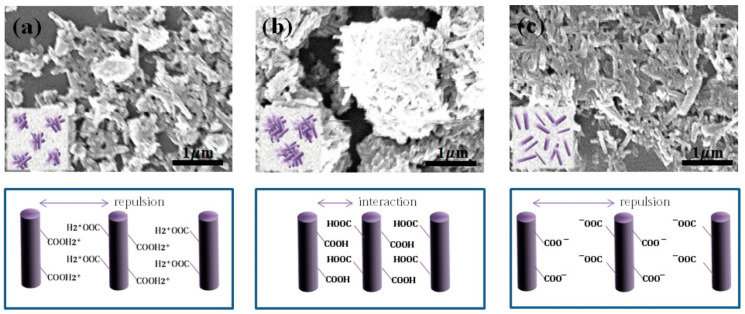
SEM images and a schematic illustration of HNTs-COOH in dry form from (**a**) acidic (pH 1), (**b**) neutral (pH 7), and (**c**) alkaline solution (pH 12). Reproduced with permission from [128].

In particular, by SEM investigation they observed that in neutral solution the HNTs-COOH nanomaterials were highly aggregated, probably as a consequence of some attractive interactions among the different organic groups on HNTs’ surface. On the contrary, in acidic and alkaline conditions, the tubes were more dispersed, due to the presence of repulsive interactions (carboxylic groups are fully protonated or deprotonated at pH 1 and 12, respectively) (Figure 12).

**Figure 13:**
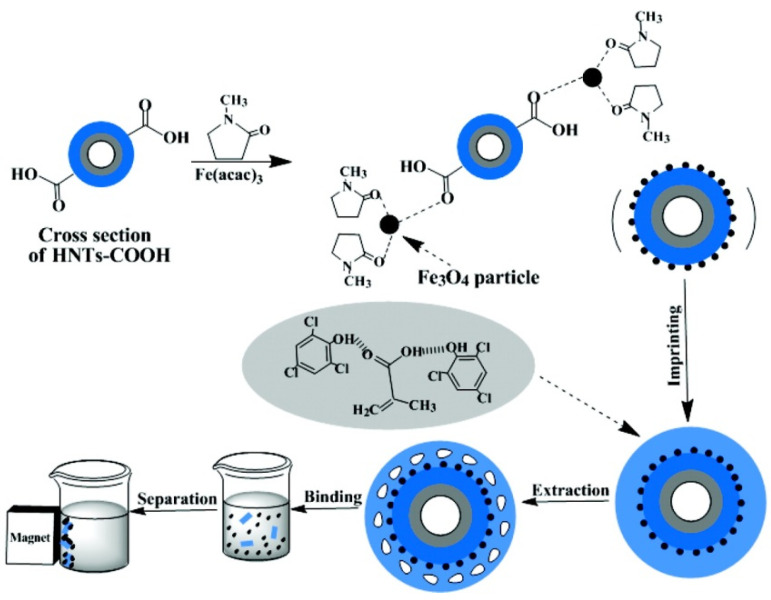
Synthesis of molecular imprinted polymers (MMIPs) and their application for removal of 2,4,6-TCP (trichlorophenol) with the help of an applied magnetic field. Reproduced with permission from [129].

The same nanomaterial was used in 2011 by Yan et al. to synthetize an innovative sorbent for 2,4,6-trichlorophenol (TCP) [129]. Starting from HNTs-COOH, the authors of this work attached on HNTs; surface magnetic nanoparticles via a robust linkage obtained by the thermal decomposition of an organic precursor such as Fe(acac)_3_ (Figure 13). This was used as support for the synthesis of molecular imprinted polymers (MMPIs). It exhibited excellent specific recognition, thermal stability, saturation magnetization, and more importantly it was easily separated from the suspension by an external magnetic field. As sorbent, the material showed a fast and selective recognition of TCP from aqueous solutions.

Some years later, by exploiting the carboxylic groups onto HNTs’ external surface, Liu et al. grafted, via *N*-(3-dimethylaminopropyl)-*N*′-ethylcarbodiimide/*N*-hydroxysuccinimide reaction, chitosan to HNTs, forming the HNTs-g-chitosan hybrid [130]. This hybrid was employed for the immobilization of curcumin by supramolecular interactions, and was successful used as anticancer agent. The authors found that the covalent grafting of chitosan on HNTs increases hemocompatibility, stability of the clay in body liquid, loading efficiency of curcumin, and cytocompatibility. Simultaneously, the same authors used the HNTs-g-chitosan hybrid developed as carrier for doxorubicin [131]. As far the drug release is concerned, two different behaviors were observed very slowly in PBS solution (only 6.40% was released after 45 h); this was faster in cell lysate where 61.9% of the amount released was observed after 12 h. In this case it was possible to achieve a sustained and selective release of the doxorubicin in a “tumoral environment”.

In 2018, HNTs-COOH was used to attach a 6-arm PEG-NH_2_ polymer onto HNTs, which was further modified by attachment of carbon quantum dots for additive fluorescent properties and by conjugation of biotin molecules to achieve targeting for the tumor cells [132]. The synthetized delivery system showed an higher loading efficiency towards quercetin with respect to pristine HNTs and a pH controlled release of the drug over time.

Tartaric acid was covalently linked on HNTs’ surface by an amide condensation between its carboxylic groups and HNTs-NH_2_. This system was subjected to pyrolysis by microwave irradiation at 240 °C with a MW heating time of 3 min, in the presence of ethylene diamine as passive agent, to obtain carbon nanodots (CDs) covalently linked on HNTs (HNTs-CDs) [133]. This procedure allowed synthetized, small, and uniformly sized carbon nanodots thanks to the presence of HNTs which acted as template and, more importantly, it rendered the inorganic HNTs a luminescent material. Fluorescent experiments both in solution and in solid state showed that HNTs-CDs emitted in different regions of the electromagnetic spectrum depending on the irradiation wavelength. A year later the system was used as fluorescent probe for application in gene therapy. The system showed high propensity to interact with calf thymus DNA, chosen as model [13].

### 3.2. Thiol Modified HNTs (3-Mercaptopropyltrimethoxysilane Grafting)

In 2014, for the first time, a silane was introduced bearing a thiol terminal group on HNTs’ surface. In one example, 3-mercaptopropyltrimethoxysilane (MPTMS) was grafted onto HNTs’ external surface by heating at reflux in the presence of a toluene HNTs dispersion for 24 h. The HNT-SH nanomaterial was then oxidized by means of hydrogen peroxide, to obtain a HNT-SO_3_H [134]. The latter procedure was needed to synthetize an acid-chromic chloride bi-functionalized catalyst for the one-pot conversion of cellulose to 5-hydroxymethyl furfural in ionic liquid as solvent. Independently, the grafting of MPTMS was investigated by microwave irradiation. It was found that by adopting MW as heating source, it was possible to reach comparable loading in a lesser time (only 1 h of irradiation) in comparison to the traditional heating [135]. This system was used as scaffold to attach to HNTs the so-called supported ionic liquid phase (SILP) and subsequently used for immobilization of palladium nanoparticles. The chosen ionic liquid was 1-vinyl, 3-octyl imidazolium bromide, which was attached to HNTs-SH by a thiolene reaction. Afterwards Pd nanoparticles were supported on the nanomaterial by ion exchange with PdCl_4_^−^ followed by NaBH_4_ reduction obtaining a HNTs-SILP@Pd catalyst.

The HNTs-SILP@Pd was used as catalyst for the Suzuki cross coupling between phenylboronic acid and a series of aryl halide. The system showed good recyclability up to five cycles [136]. Two years later, it was demonstrated that the synthetized HNTs-SILP@Pd did not show any phytotoxic effects on the growth of Raphanus sativus L. [137].

**Figure 14:**
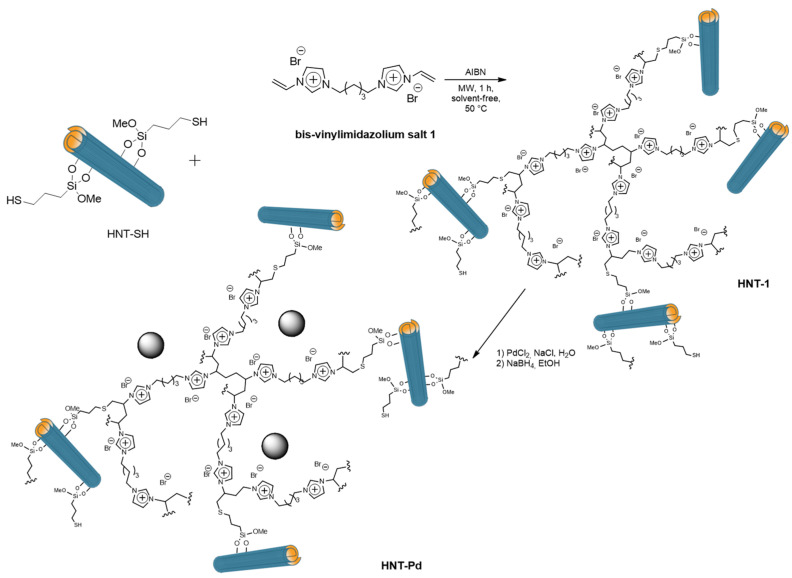
Schematic representation of the synthesis of HNTs-Pd catalyst.

In 2018 a step forward the use of HNTs-SILP materials for catalytic application was made by developing HNTs modified with highly cross-linked imidazolium salts. HNTs-SH was reacted with bis-vinyl-imidazolium salts under MW irradiation, in the presence of AIBN and in solvent-free conditions, obtaining the HNTs-Pd material in Figure 14 [138]. After immobilization of PdNPs it was tested as catalyst in the Suzuki and Heck reactions under MW irradiation. The high catalytic performances of these materials were highlighted by the high TONs (up to 194,000) and remarkable TOF values (up to 3,880,000 h^−1^) obtained. Furthermore, the catalyst showed good recyclability for at least ten cycles with negligible Pd leaching. Finally, ICP-OES measurements highlighted that negligible palladium leaching occurred in the final products, whose value is lower than that allowed by current government legislation.

In 2014 was also reported the possibility of grafting onto HNTs external surface cyclodextrin units to develop multicavity systems. Starting from thiol modified HNTs, a thiolene reaction mediated by AIBN, with allyl modified cyclodextrin was carried out [139]. The nanomaterial obtained showed an enhanced aqueous stability in comparison to pristine HNTs, and two cavities available for interaction with drugs. Preliminary adsorption investigations toward curcumin molecules, chosen as model, showed that curcumin can be loaded only in the cyclodextrin cavity. In addition, the presence of unmodified allyl groups onto the secondary rim of the cyclodextrin units allowed the post-modification of the system by the linkage with three different sugars; namely, mannose, galactose and lactose. Two years later the obtained nanomaterial was investigated for the co-delivery of two different drugs, i.e., curcumin, which interacted with the cyclodextrin cavity and silibinin loaded mainly in HNTs’ lumen [140]. Enzyme-linked lectin assays (ELLA) demonstrated that, for instance, the highly mannoside–cyclodextrins HNT entities displayed high affinity towards mannose selective ConA lectin, which promoted cellular uptake, as confirmed by fluorescence microscopy. In particular, it was found that the material penetrated cell membrane, and more specifically it crossed nuclear barriers surrounding the cell nuclei. Biological assays showed that the new drug delivery system exhibited anti-proliferative activity against two cancer cell lines.

In the meantime, the cyclodextrin rim of the system HNTs–cyclodextrin was post-modified by different thiols to obtain an amphiphilic system for the co-delivery of highly hydrophobic natural molecules [141]. In this case, the authors investigated the delivery of quercetin, loaded in the cyclodextrin core, and silibinin loaded into HNTs. Once again, biological results showed promising antiproliferative activity as a consequence of synergistic effects among the two molecules.

**Figure 15:**
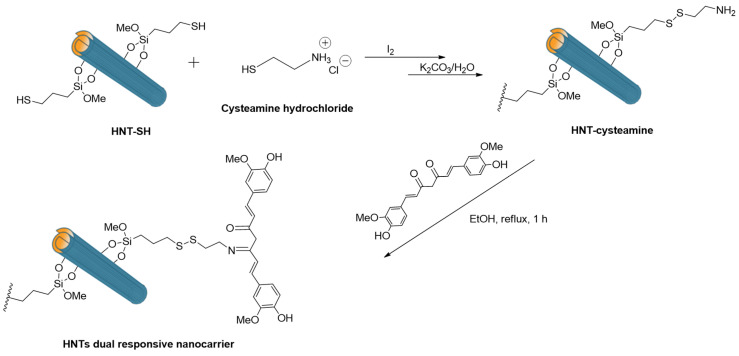
Schematic representation of the synthesis of the dual responsive nanocarrier based on HNTs.

Thiol groups on halloysite nanotubes were important for the development of a dual-responsive nanocarrier for curcumin based on HNTs [142]. To reach this objective, HNTs-SH was reacted with cysteamine molecules giving rise to an HNT scaffold bearing a glutathione (GSH)-responsive bond (Figure 15); i.e., a disulphide bond. Afterwards, curcumin was covalently linked to this material via a pH-responsive imine bond. The dual stimuli-responsive nature of the nanocarrier was studied via kinetic experiments in different conditions. It was found that the curcumin release occurred only in acidic medium as a consequence of the hydrolysis of imine bonds. On the contrary, the molecule is released in physiological conditions only after the addition of glutathione, confirming the redox responsive nature of the disulphide bond.

**Figure 16:**
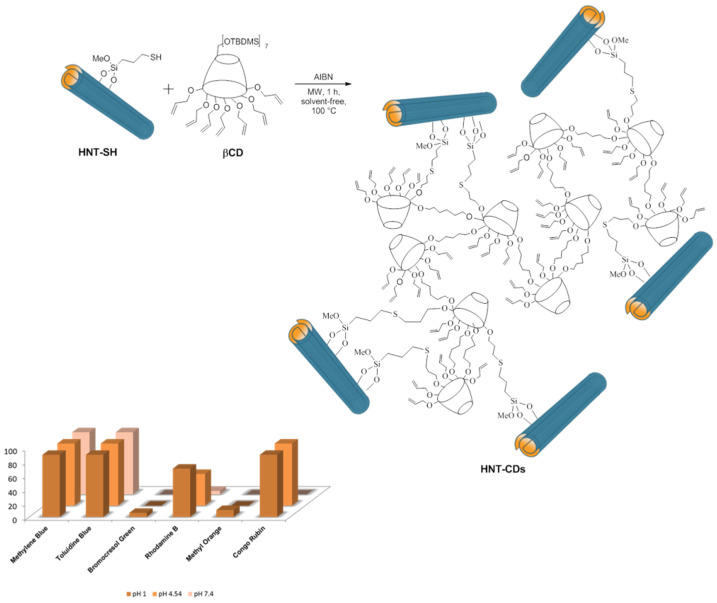
Schematic representation of the synthesis of HNTs and cyclo-desxtrin nanocomposite as adsorbent for organic dyes. Adapted with permission from [143].

The thiol modification of a different type of HNTs, in particular the one mined in New Zealand, led to a lower loading percentage of organic modification with respect to other HNTs (1 wt% and 2.1 wt% for New Zealand HNTs and other types, respectively). This result was explained by the high purity grade of the New Zealand HNT and therefore minor defects on its surface, and less Si-OH groups available for the chemical modification. In any case, by exploiting this different loading, it was possible to graft cyclodextrin units, obtaining organic-inorganic nano-sponges [143]. These nanocomposites showed both HNTs and cyclodextrin features and an hyper-reticulated nature which was exploited for the adsorption of organic dyes (Figure 16). The experimental results obtained highlighted the nano-sponges’ selective adsorption capacity toward cationic dyes, with respect to the anionic dyes from aqueous solution.

A year later, the post-modification of the above reported system allowed the authors to obtain a mucoadhesive system for the delivery of clotrimazole for vaginal or buccal treatment of Candidiasis [144]. Recent studies of this material showed that it could be a good candidate to deliver polyphenolic compounds [145].

By exploiting the strong gold-thiol affinity, in 2019 the HNTs-SH system was used for the synthesis of gold based catalyst [146]. The formation of the AuNPs was achieved by adding an aqueous solution of HAuCl_4_ to an aqueous dispersion of HNT-SH, followed by reduction with NaBH_4_ in methanol. The catalytic performances of the HNTs@Au were evaluated in the reduction reaction of 4-nitrophenol to 4-aminophenol. The results showed that HNT@Au possessed outstanding catalytic activity, reaching remarkable turn over frequency (TOF) values (up to 2,204,530 h^−1^).

**Figure 17:**
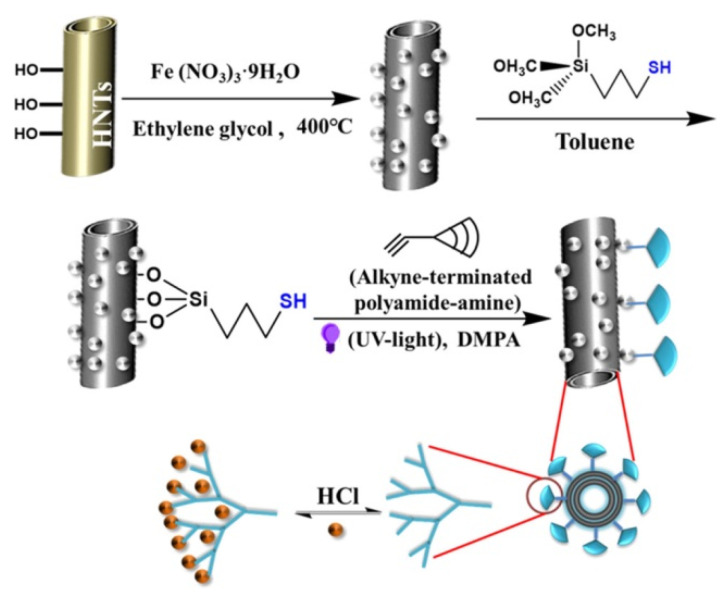
Schematic diagram showing the synthetic route of PAMAM grafted magnetic halloysite and removal of Pb(II) from water. Reproduced with permission from [147].

More recently, thiol modified magnetic halloysite was subjected to a thiolene click reaction with terminal alkyne PAMAM units to form a dendrimer modified HNT with high amino group density for water remediation (Figure 17) [147]. The nanomaterial was used as sorbent for Pb^2+^ ions demonstrating a high adsorption capacity of 194.4 mg g^−1^. Recyclability studies showed that the nanomaterial synthetized still possessed 90% of the maximum adsorption capacity after six consecutive runs.

### 3.3. Vinyl Modified HNTs (KH570 Silane Grafting)

In the 2000s the attention of researchers was mainly focused on finding novel methods to improve the interfacial properties between HNTs and polymeric matrices. After 2008, when APTES was covalently grafted onto HNTs, a novel research line was started based on covalently modified HNTs dispersed into different polymers. The organo-silane often chosen for this purpose was *γ*-methacryloxy-propyl trimethoxy-silane (KH570), which, due to its terminal group, ensured better affinity with polymers. For example, in 2010, halloysite nanotubes were modified by KH570 to improve their dispersion in ethylene propylene diene monomer (EPDM) [148]. The authors of this work found that the tensile strength and tensile modulus at 100% elongation of the nanocomposites were higher in comparison to those of EPDM/unmodified HNTs while the elongation at break decreased a little after modification of the HNTs. In addition, SEM and TEM studies revealed the better dispersion of the modified HNTs in the EPDM matrix. Similarly, in 2012, KH570 modified HNTs led to the formation of polylactide (PLA)/HNTs nanocomposites which showed better thermal stability, morphology with higher nanofiller contents, and good tensile stress characteristics, associated with higher impact strength (3.5 kJ m^2^ by addition of 12% HNTs, with respect to 2.8 kJ/m^2^ for pristine PLA) [149].

**Figure 18:**
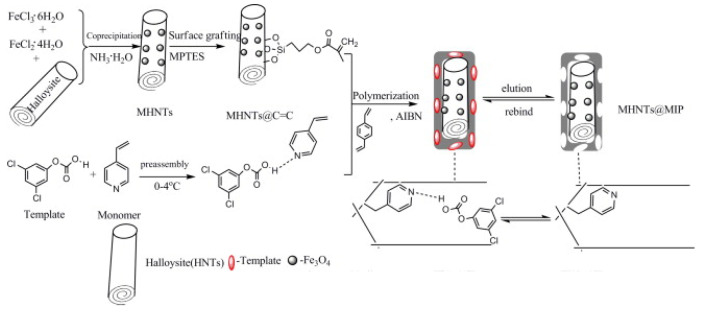
Schematic representation of preparing MHNTs@MIP. Reproduced with permission from [150].

In 2014, 3-methacryloxypropyltrimethoxy silane was grafted onto Fe_3_O_4_@HNTs (MHNTs) to provide a carbon-carbon double bond on the HNTs’ surface for the synthesis of molecularly imprinted polymer based on HNTs (MHNTs@MIP) [150]. It was synthetized by a precipitation-polymerization method, using vinyl-modified MHNTs as support material, 2,4-dichlorophenoxyacetic acid (2,4-d) as template, 4-vinylpyridine as the functional monomer, divinyl-benzene as the crosslinker, and 2,2-azobisisobutyronitrile as the initiator (Figure 18). 2,4-d was chosen as template, because, due to its extensive use as herbicide and its toxic nature, it is necessary to develop a valuable method for its identification and separation. Once the 2,4-d is removed from the MHNTs@MIP, a cavity was present in the imprinted polymer which was exploited for the adsorption of the herbicide. MHNTs@MIP displayed excellent characteristics, such as repeated use, high binding capacity, and fast mass transfer for 2,4-d.

Similarly, the same scaffold, i.e., 3-(trimethoxy-silyl)-propyl-methacrylate grafted onto magnetic halloysite, was used for the synthesis of imprinted polymers with a shell thickness of 35 nm for the removal of tetracycline [151].

In 2015, SBR matrix was filled with 27 wt % of *N*-isopropyl-*N*’-phenyl-p-phenylene-diamin (antioxidant 4010NA) loaded into vinyl modified HNTs to provide a rubber aging protection [152]. The silanization of HNTs allowed their excellent miscibility with the rubber matrix. Encapsulating the antioxidant in the nanotubes allowed for an antioxidant concentration of 3.2 wt % (which otherwise would cause rubber defects) without any indication of surface blooming. The release of 4010NA from HNTs both in nonpolar cyclohexane and in water was observed from 4 h to days, but after inserting the tube-encapsulated antioxidant into bulk rubber it was extended to more than 9 months (66 wt % was released in the first five months). After the incubation at 90 °C for seven days, the SBR and antioxidant-loaded halloysite composite showed well-retained mechanical properties when compared to a control sample.

### 3.4. Azido Modified HNTs (Grafting of 3-azidopropyltrimethoxysilaneAzidopropyltrimethoxysilane)

3-azidopropyltrimethoxysilane was, for the first time, loaded onto HNTs’ external surface in 2014 to introduce terminal groups available for the synthesis of triazole modified HNTs by the azido-alkyne CuAAC-type coupling reaction. After the synthesis of triazole rings, they were quaternized by alkylation with iodobutane to obtain HNTs modified with triazolium salts [153]. The presence of these salts on the external surface of the nanomaterial gave rise to a prodrug system which could be easily taken up by cells due to the presence of a positive charge. The system was employed for the delivery of curcumin molecules, and the antiproliferative activity was tested, by means of MTT test, on different cancer cell lines. The obtained results highlighted the importance of the triazolium salts since they possessed a cytotoxic activity with an IC_50_ of 40.0 ± 14.6 mM. Furthermore, triazole moieties on HNTs showed synergistic effects with the loaded curcumin molecules, increasing the cytotoxic performance.

A year later, the same authors reported the use of the same system for the delivery of cardanol [154]. In this case, the drug molecules were found to be mainly adsorbed on the external surfaces of the HNTs’ nanomaterials. Similarly to curcumin, the cardanol adsorbed on HNTs’ system possessed increased cytotoxic effects in comparison to free cardanol in physiological conditions, as a consequence of an increased bioavailability of the drug and the combination with the triazolium salt groups covalently grafted onto HNTs.

**Figure 19:**
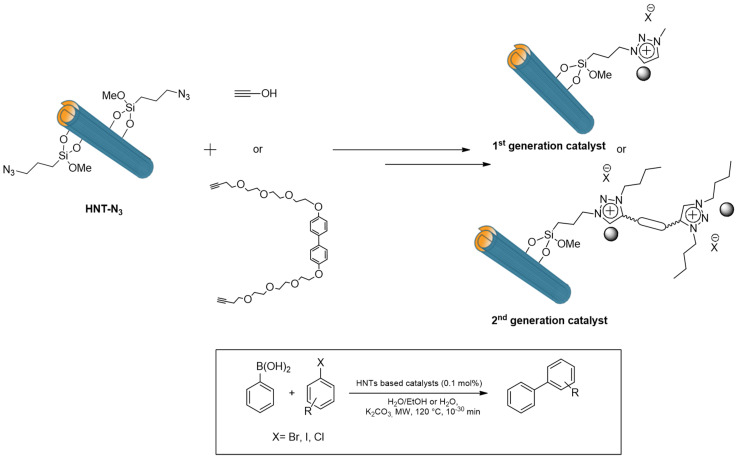
Synthesis of the first and second generation HNTs triazolium salt based catalysts and their application in the Suzuki reaction.

In 2015, azido modified HNTs were used as scaffold for the synthesis of first- and second-generation heterogeneous catalysts, based on triazolium salts, for the stabilization of palladium nanoparticles [155]. In particular, the development of dicationic triazolium salts based on HNT systems allowed the authors to obtain high Pd loading and good thermal stability. The nanomaterials were used as catalyst in the Suzuki cross coupling reactions between phenyl boronic acid and several aryl halides showing promising catalytic activity in water under MW irradiation (Figure 19).

More recently, curcumin molecules were linked to HNT-N_3_ nanomaterials with different terminal groups, obtaining multifunctional systems for anticancer therapy [156]. In detail, curcumins with one or two terminal alkyne groups were reacted by a click reaction with HNTs-N_3_, forming nanocarriers with a single curcumin unit or a curcumin bridge between two HNTs depending on the starting organic molecules. Subsequently the obtained carriers were loaded with other curcumin molecules or with doxorubicin to reduce the side effects often associated with the use of the latter drug. Cytotoxic measurements on two triple negative breast cancer cell lines, SUM 149 and MDA-MB-231 and on two acute myeloid leukemia cell lines, HL60 and its multidrug resistant variant HL60R, showed that the antiproliferative activity of the system is strictly correlated both to the nanoparticles’ size and to the release profile of the drug supra-molecularly loaded on HNTs’ surfaces.

### 3.5. Miscellaneous

Amino-silanes bearing two or three amino groups, respectively, were used by Sepúlveda-Escribano et al. in 2011, to obtain HNT based supports for the heterogeneous atom transfer polymerization of methyl methacrylate (MMA) into poly(methyl-methacrylate) (PMMA) using CuBr as catalyst [157]. The anchored silanes can act as a ligand for CuBr as well as a catalyst for the heterogeneous polymerization. Furthermore, the authors reported that by grafting onto HNTs surface silanes with three amino groups it was possible to obtain a better control of the polymerization compared to the silane with two amino groups. This is due to hydrogen bonding between amino groups in the silane and OH groups present in the surface of halloysite after being grafted with silane.

In the same year, *N*-β-Aminoethyl-γ-aminopropyl trimethoxy-silane (KH-792) was grafted onto HNTs’ surface in order to develop a sorbent material for Cr(VI) removal from wastewater [158], exploiting the high affinity of amino groups towards metal cation. The experimental results showed that electrostatic interaction between adsorbent and Cr(VI) played an important role in the process of adsorption.

Going further, the same authors used the KH-792 grafted HNTs as scaffold to anchor, by in situ polymerization, poly(4-vinylpyridine) on HNTs, obtaining a solid support for Ag nanoparticle immobilization [159]. Once synthetized, the nanomaterial was introduced in polyether-sulfone (PES) ultrafiltration membranes to synthetize a novel nanocomposite with antibacterial and antifouling properties.

Similarly, *N*-(2-aminoethyl)-3-aminopropyltrimethoxysilane was grafted onto HNTs and subsequently the amino groups were subjected to quaternarization by alkylation. This system was used by Jiang et al. as support for {PO_4_[W(O)(O_2_)_2_]_4_}^3−^ and employed as catalyst for the epoxidation of soybean oil [160].

**Figure 20:**
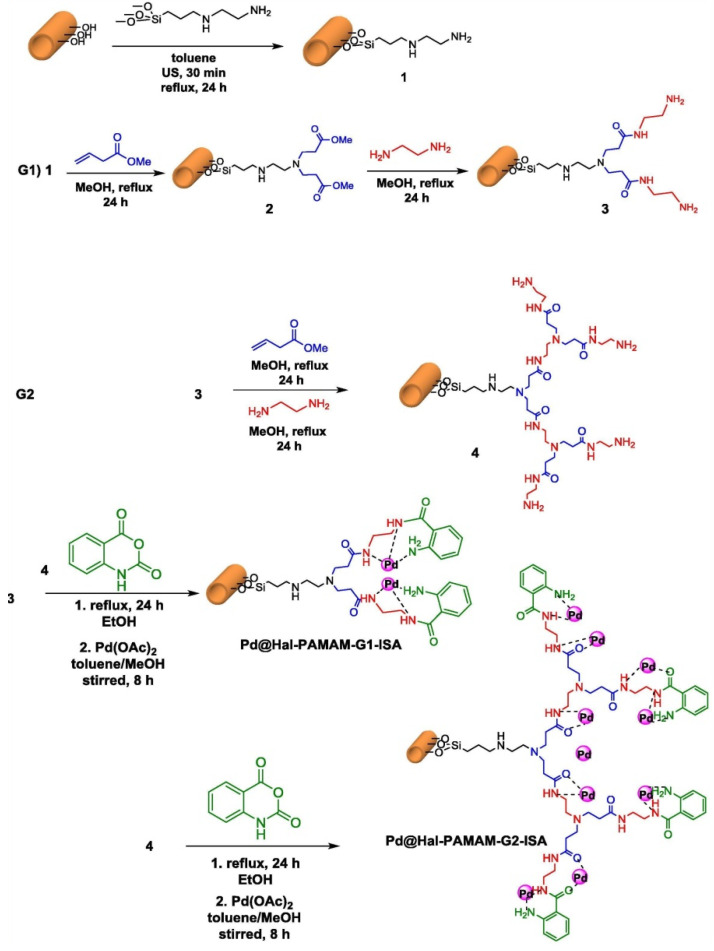
General procedure for the synthesis of the Pd@HNTs-PAMAM-ISA. Reproduced with permission from [161].

Recently, the same silane, was grafted onto the HNTs’ surface and used as scaffold for the construction of PAMAM-like dendrimers (first and second generation, G1 and G2, respectively) which were further modified by linking them to isotoic anhydride [161]. The subsequent treatment with Pd(OAc)_2_ in toluene followed by reduction with NaBH_4_ gave rise to Pd@HNTs-PAMAM-ISA materials (Figure 20). These were used as catalysts in the Sonogashira and Heck reactions in a mixture of water/ethanol 1:1, showing promising catalytic activity and recyclability up to 10 cycles with slight loss of the catalytic activity and Pd leaching.

3-Chloropropyltrimethoxysilane was grafted onto HNTs and used as scaffold to attach poly(4-vinylpyridine) (P4VP) brushes via reverse atom transfer radical polymerization [162]. In this way, the authors introduced onto HNTs several pyridine rings which are able to immobilize Cu nanoparticles. The nanomaterial so obtained showed good antibacterial activity against Escherichia coli.

In the same year, following a similar approach, amphiphilic brushes consisting of P4VP and polystyrene (PS) segments were grafted onto HNTs, via reversible addition-fragmentation chain transfer polymerization (RAFT) [163]. To reach this goal, the nanotubes were first modified with 3-glycidoxypropyltrimethoxysilane and, after refluxing in a methanolic solution of HCl, the RAFT agent, needed for the polymerization, was added. The final material, possessing both P4VP and PS, could emulsify water/soybean oil diphase mixture and the emulsification performance was dependent on the microstructure of amphiphilic brushes, such as hydrophilic/hydrophobic segment size and sequence.

**Figure 21:**
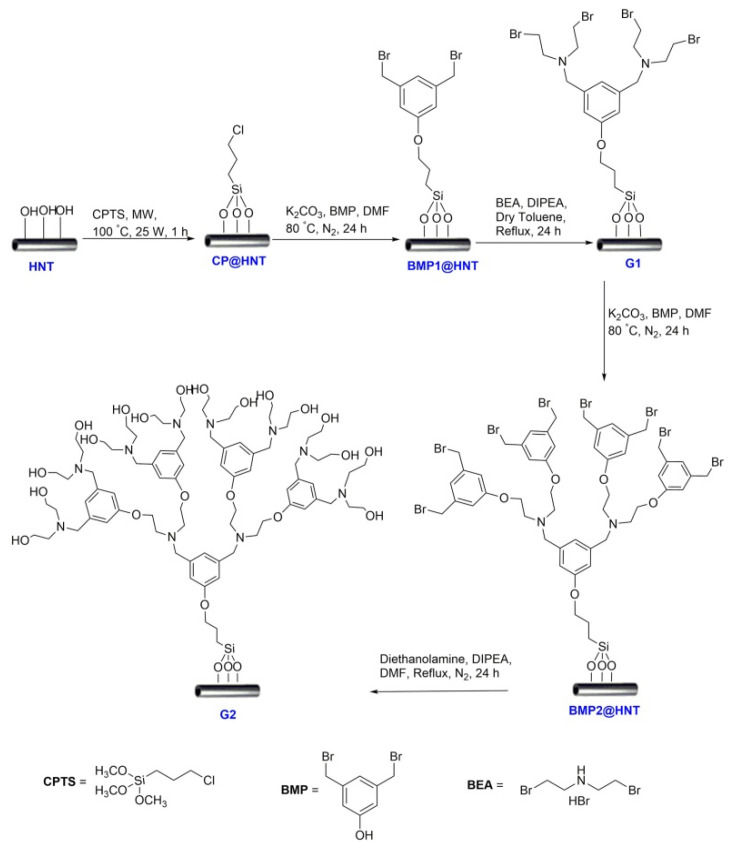
Preparation of halloysite nanotube–functionalized dendrimer. Reproduced with permission from [164].

Recently, HNTs-Cl nanomaterial was used as starting material for the development of dendrimers which can efficiently stabilize Ce(IV) for catalytic applications (Figure 21) [164]. The catalyst was used for the synthesis of a series of pyrido[3,2-c]coumarins via a one-pot, single step, three-component reaction of 4-aminocoumarin, aryl ketones, and aldehydes. The results showed that in these conditions it was possible to obtain efficient regioselective synthesis of pyrido[3,2-c]coumarins from internal and terminal alkynes.

Zhong et al. used tetra-ethoxy-silane (TEOS) and octyl-triethoxysilane (OTES) to modify the external HNTs’ surface in order to render it more hydrophobic than that of pristine HNTs [165]. After loading of ibuprofen molecules, due to the organic modification it was possible to tune the drug release up to 100 h.

**Figure 22:**
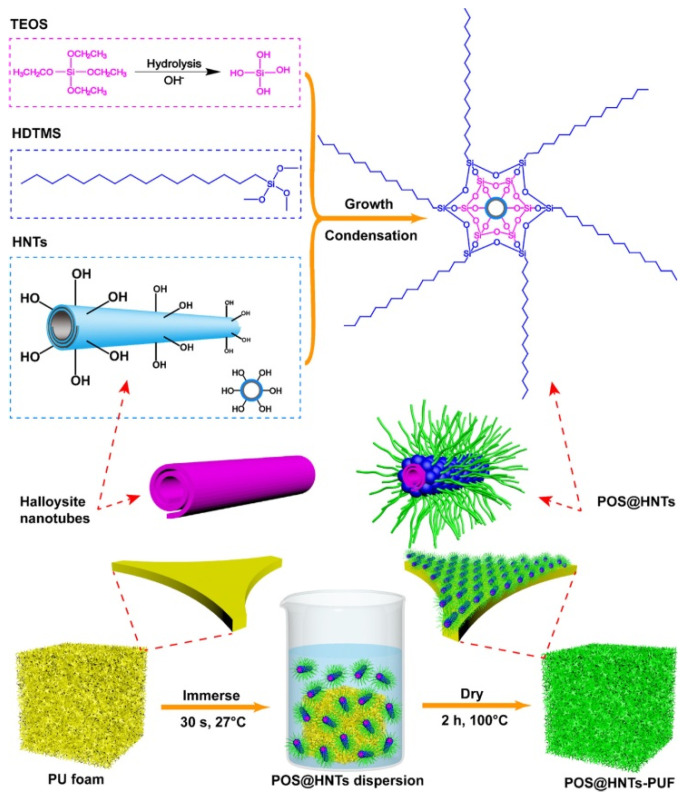
Preparation and reaction mechanism of the superhydrophobic POS@HNTs coated polyurethane foam. Reproduced with permission from [167].

Some years later, a similar approach was reported by Liu et al. who modified the HNTs’ surface by hydrolytic condensation of n-hexa-decyl-trimethoxy-silane (HDTMS) and tetra-ethoxy-silane (TEOS) to obtain poly-siloxane modified HNTs (POS@HNTs) [166]. A superhydrophobic coating with high water contact angles, ultralow sliding angles, oil/water separation, and self-cleaning functions was prepared by spray-coating a POS@HNTs suspension onto various substrates. The authors of this work developed a material which can find application in waterproof materials, self-cleaning coatings, and oil/water separation devices, since they found that, for example, the POS@HNTs coated meshes efficiently separated oils from water with high efficiency. The same authors reported that the use of the POS@HNTs were assembled on polyurethane foam (PUF) by a dip coating process (Figure 22) [167]. The modified foam showed highly selective absorption of oils and organic solvents. The POS@HNTs-PUF, indeed, adsorbed 105 times of its own weight in chloroform and maintained its absorbency after 10 times repeated use (soak-squeeze cycles). Furthermore, the POS@HNTs-coated PUF automatically extinguished itself, preventing the melted fragments from igniting other flammable materials.

**Figure 23:**
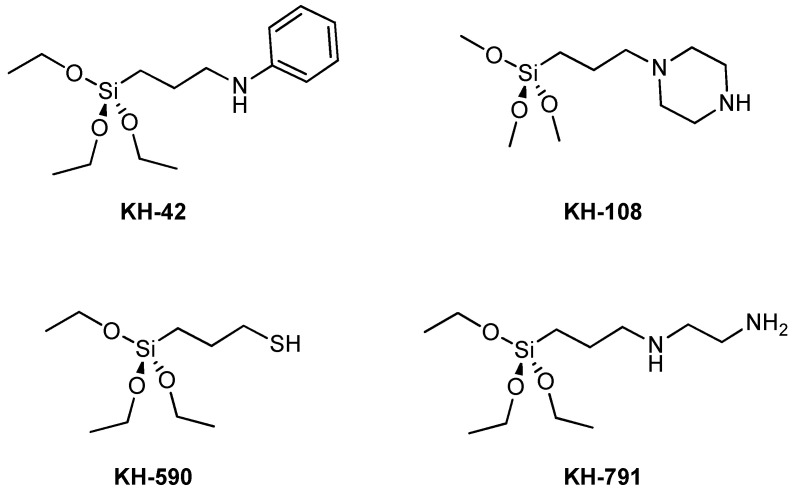
Silanes used for the modification of HNTs for environmental purposes.

In 2017, Wang et al. exploited the possibility of using different silane to modify the surface of magnetic halloysite to obtain adsorbent for the removal of metal cation [168]. The silanes chosen, reported in Figure 23, possess the peculiarity of bearing different terminal functionalities which can interact in different ways with the metals.

The adsorption efficiency was tested for the simultaneous removal of Cr(VI) and Sb(V) from simulated wastewater.

The experimental findings showed that the KH-42 modified HNTs possessed the highest removal efficiency towards Cr(VI) which can be attributed to the interactions between HCrO_4_^−^ and aromatic compounds on HNTs, forming anion-π complexes. Conversely, KH-108 modified HNTs showed the highest removal efficiency for Sb(V) at acidic conditions, which might be due to the interaction between negatively charged Sb(OH)_6_^−^ and the positively charged amino functional groups present in an acidic environment.

**Figure 24:**
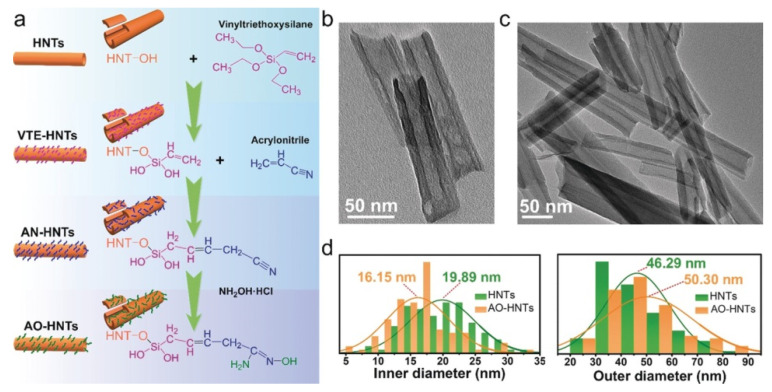
Functionalization of HNTs. (**a**) Illustration of the fabrication of amidoxime-HNTs; TEM images of (**b**) HNTs and (**c**) amidoxime-HNTs; (**d**) inner and outer diameter distribution of HNTs and amidoxime-HNTs. Reproduced with permission from [169].

Recently, HNTs were modified with vinyl-trimethoxy-silane with the final goal of introducing acrylonitrile on HNTs’ surface [169]. Then, it was further amidoximated into amidoxime group, to fabricate an adsorbent for uranium extraction (Figure 24). The adsorbent showed a uranium adsorption capacity of 456.24 mg·g^−1^ in 32 ppm uranium-spiked simulated seawater with a saturation time of 13 h. In natural seawater, the material reached the uranium extraction capacity of 5.70 mg·g^−1^ and 9.01 mg·g^−1^ after field tests of 5 days and 30 days, respectively.

## 4. Modification of the Inner Lumen

Unlike silane grafting on the silanol groups present on HNTs’ external surface, the covalent modification of the HNTs’ lumen is not so common. Up to now, indeed, to the best of our knowledge, only few examples are reported in the literature. The grafting procedure in this case foresees the selective hydrophobization of the halloysite inner surface by reaction with suitable organic compounds. The first work reported was in 2012 by Lvov et al. who explored the grafting of octadecyl phosphonic acid on the hydroxyl aluminol groups of the inner lumen. They found that the reaction occurred selectively in the lumen and no bonding on the siloxane outer tube surface was observed. The obtained materials, presenting a hydrophobic core, were successfully used as sorbent for hydrophobic molecules such as bisphenol-bis(diphenyl phosphate) or ferrocene [170,171]. Furthermore, the hydrophobic modification allowed a slower release of the active agents from the lumen with respect to the non-modified halloysite.

In the same year, the same authors investigated the possibility of linking catechol based compounds to the inner HNTs’ surface. They demonstrated that, for instance, 2-bromo-*N*-[2-(3,4-dihydroxyphenyl)ethyl]-iso-butyryl amide, a catechol derivative, can be covalently linked in aqueous conditions to the alumina innermost surface but not to the silica outermost surface of halloysite [12].

Some years later, Wu et al., grafted phenyl boronic acid onto alumina groups into HNTs’ lumen and used the resulting nanomaterial as fluorescence probe for the detection of hydrogen peroxide (H_2_O_2_) at low levels, which might provide potential applications in the biomedical field [172].

**Figure 25:**
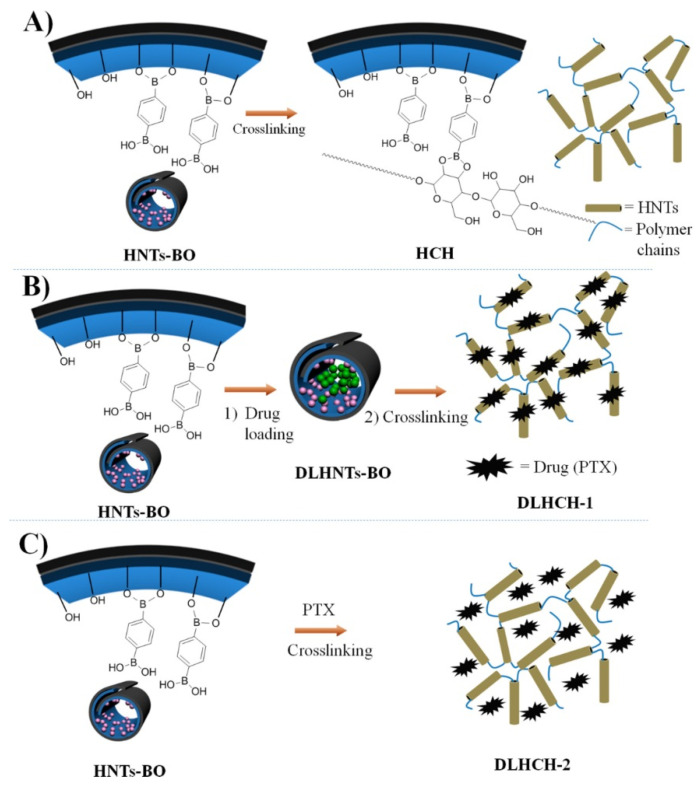
Preparation of HNT-based hydrogels. (**A**) The route and mechanism of preparing HCH; (**B**) preparation of DLHNTs-BO and DLHCH-1 (PTX was loaded in the lumen in DLHNTs-BO and DLHCH-1); (**C**) preparation of DLHCH-2 (PTX was embedded in the matrix network in DLHCH-2). Reproduced with permission from [173].

In 2017, the same authors [173] exploited the reaction between 1,4 phenylene-bisdiboronic acid and HNTs to obtain an efficient system to immobilize compressible starch by reaction of the vicinal diol in the carbohydrate and the arylboronic acid units on the HNTs’ lumen, forming a hydrogel (Figure 25). The system was used for the loading of pentoxifylline, a model drug, which is released form the carrier in the presence of an inflammation since the hydrogel is disrupted in the presence of H_2_O_2_.

Dedzo et al. reported the formation of specific Al–O–C bonds on the HNTs’ inner lumen by grafting of ionic liquids onto aluminol inner groups [174]. Due to this strategy, the authors obtained a nanocontainer for catalytic purposes by immobilizing Pd nanoparticles onto the ionic liquids’ modified HNTs. The catalytic performances were evaluated in the reduction reaction of 4-nitrophenol to 4-aminophenol.

## 5. Intercalation

When the research on HNTs started, the main problem was how to differentiate between kaolinite and halloysite—7 Å. It was known that HNTs showed greater propensity for intercalating small organic molecules than does kaolinite. Therefore, intercalation became an important tool to identify and quantify HNTs—7 Å and kaolinite in mixture. The first study on the intercalation properties of HNTs is attributed to Joussein et al. who reported in 2007 a detailed physico-chemical characterization of formamide intercalated HNTs [175].

Subsequently, Wypych et al. [176] studied the intercalation of urea using the Fe(III) ion’s impurity as an EPR probe to study the thermal stability of intercalated urea-halloysite material, and to analyze the modification of the structure of the intercalation compound in response to heat treatments. The authors found that the intercalated urea increased the structural order in HNTs. Furthermore, by heating the intercalated HNTs up to 200 °C, it was possible to remove urea, reestablishing the original value and crystal structural disorder between the HNT layers. This aspect was considered crucial because the material could be used in several industrial applications.

In 2011, Khelifa et al. reported the intercalation of sodium acetate into HNTs at different contact times [177]. They demonstrated by XRD measurements that the fraction of halloysite intercalated with sodium acetate linearly increased with contact time and reached a high degree of intercalation, i.e., 90% with basal spacing remaining constant at 10.1 Å. The intercalation of small organic molecules into halloysite led to an increase of HNTs’ adsorption properties towards metal ions. The study highlighted that the intercalated HNTs showed a retention of the loaded Cu(II) metal 2.2 times larger than pristine clay.

Intercalated HNTs with phenyl-phosphonic acid were successfully used as additives of an epoxy to form partially and fully intercalated nanocomposites [178]. It was found that better dispersion in the epoxy was achieved using the unfolded and intercalated halloysite than using the pristine halloysite. There was a significant increase in fracture toughness for the epoxy composites with unfolded and intercalated halloysite particles, without sacrificing other properties such as strength, modulus, glass transition temperature and thermal stability.

## 6. Chemical Manipulation

In order to improve the physico-chemical properties of HNTs, in particular to improve the loading capacity, several chemical approaches have been investigated. Exploiting the different chemical composition of inner and outer surfaces, it was possible to selective remove one layer or another by working in different pH solutions.

Bavykin et al. reported for the first time the long-term stability of halloysite in 0.001–1 mol·dm^−3^ solutions of different acids (H_2_SO_4_, HCl and acetic acid), a strong base (NaOH) and distilled water [179].

**Figure 26:**
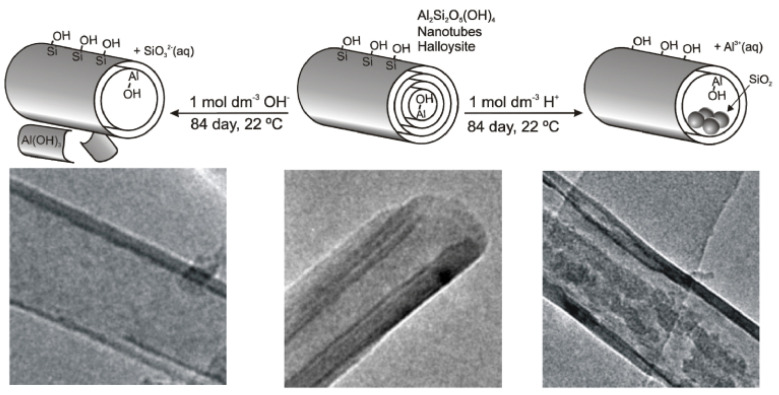
Scheme of transformation of halloysite (Al_2_Si_2_O_5_(OH)_4_) nanotubes in strong acid and alkaline solutions leading to formation of amorphous nanoparticles of SiO_2_ and amorphous nanosheets of Al(OH)_3_, respectively. Reproduced with permission from [179].

The study showed that halloysite was kinetically stable in water and in weak organic or dilute (1 mmol·dm^−3^) inorganic acid and alkaline solutions at room temperature. Conversely, in strong acid solutions, the greater solubility of Al(III) versus Si(IV) leads to the formation of small SiO_2_ nanoparticles inside the tubes, enhancing the surface area and pore volume of the sample. In concentrated NaOH solutions the greater solubility of Si(IV) versus Al(III) leads to the formation of fragmented flaky particles containing layers of Al(OH)_3_ (Figure 26).

Similarly, Chen et al. [180] reported that sulfuric acid treatment of HNT destroyed its crystal structure turning it into amorphous silica. It was found that the treatment dissolved [AlO_6_] octahedral layers and induced the rupture of [SiO_4_] tetrahedral layers resulting in porous nanorods.

Lvov et al. studied the kinetic of sulfuric acid treatment of HNTs at different temperatures [181]. They found that dissolution of alumina sheets starts from inner halloysite layers and, below 70 °C, produces clay nanotubes with uniformly enlarged lumen diameter. In their opinion, alumina removal from the nanotubes took place in three steps: diffusion of hydrogen ions into the inner lumen, chemical reaction with alumina on the tube inner wall, and transport of the reaction products out of the lumen. Kinetic study revealed that chemical interaction is the rate-determining step with activation energy of 68.0 kJ/mol and pseudo-zeroth order below 70 °C. When 30–40% of dealumination is reached, the morphology of HNTs changed, according to previously reported papers. However, the enlargement of the lumen which was observed in the first step was useful in increasing the loading capacity of the nanotubes. Experiments based on the loading of corrosion inhibitors, such as benzotriazole, showed that the loading efficiency of the enlarged HNTs was four times larger than that of the pristine one.

Subsequently, acid treated HNTs were used as sorbents for a series of chloro-anilines, namely 3-,4-chloroaniline and 3,4 dichloroaniline [182]. By using different acid concentrations, ranging from 20 to 65 mass%, it was found that the modified HNTs showed the best adsorption capacity when treated with 50% sulfuric acid.

Zhang et al. investigated the adsorption capacities of acid- and heat-treated HNTs towards ofloxacin, a fluorinated quinolone, chosen as model, for biological applications [183]. In their work the authors demonstrated that the acid treatment of HNTs with HCl up to a 12 M concentration had no effects on the halloysite crystal structure and, although the tubes were shortened during the treatment, they still retained a tubular structure. The adsorption capacity of acid-treated halloysite for ofloxacin slightly decreased on increasing the HCl concentration due to the leaching of exchangeable cations and weak electrostatic interaction between the drug-Al^3+^ complexes and negative halloysite surface. On the contrary, acid-activation was useful for the release of all the adsorbed ofloxacin owing to the weakened electrostatic interaction.

Regarding heat treatment, it was found that, at temperatures below 400 °C, the HNTs did not show any variation in crystal structure; conversely, by increasing the temperature up to 500 °C, HNTs became amorphous due to the de-hydroxylation of structural aluminol groups. Similar to the acid treatment, an increase in temperature led to a decrease of the adsorption capacity for ofloxacin.

In 2015, Liu et al. reported the pre-treatment of HNTs with a piranha solution to improve the silanization reaction [68]. The use of the piranha solution to activate HNTs’ surface was advantageous compared to other widely used activation agents such as HCl or H_2_O_2_. Indeed, the so-activated HNTs showed enhanced reactivity towards APTES.

In the same year different treatments were used to modify the surfaces of HNTs and most of them envisage, as aforementioned, the use of acids. A thorough study on the effect of the strength of different acids (sulfuric, acrylic and acetic acids) on the morphology of chemically modified HNTs was proposed in 2017 by Garcia-Garcia et al. [184]. They reported that chemical treatment with strong acids such as sulfuric acid led to an aggressive etching on HNTs characterized by a highly porous structure and partial nanotube decomposition. The specific surface area of HNTs modified with sulfuric acid is of about 132.4 m^2^·g^−1^. On the contrary, the selective etching by using weak acids such as acetic and acrylic acid, led to a noticeable increase in the lumen in HNTs. In detail, the lumen diameter changed from 13.8 nm up to 18.4 and 17.1 nm for acetic and acrylic acid treatments, respectively.

## 7. Conclusions and Future Perspectives

The examples reviewed above show how the research on HNTs constantly evolves, developing smart nano-systems with tunable properties. In particular, it is reasonable to foresee that in the near future HNT based nanomaterials will be employed, for example, in actual pharmaceutical formulations for the treatment of several pathologies by an oral or topical administration of the HNT/drug systems. Up to now it has been commonly accepted that, since HNTs are mined from different sites, they could present different compositions and impurities from one mine to another. However, different studies have shown that HNTs coming from the same sites (available in tons) possess the same properties and chemical compositions. In addition, all experiments performed on them showed reproducibility of results; thus, they could be safely used in the pharmaceutical field.

Furthermore, the innovative nano-systems could be successful employed in practical industrial applications, both for the synthesis of valuable catalysts possessing a greener impact in comparison to the present ones and for the realization of nanocomposite for environmental purposes.

In order to fully exploit the physico-chemical properties of HNTs and improve some of them, they could be combined with other clays. In this context, it is reported that by mixing two different clay minerals with different properties (for example HNTs and sepiolite) a multicomponent nano-system was obtained which presents both peculiarities of HNTs and sepiolite, allowing a better and homogeneous dispersion into a cellulose matrix [185].

Recently, a novel carrier system based on HNTs as filler for LAPONITE^®^ (Lap) hydrogel for the potential intraarticular delivery of kartogenin (KGN) was proposed [186]. Due to the presence of HNTs, the gelation process was promoted, and this resulted in an improvement of the rheological properties as a consequence of electrostatic interactions between the negative HNTs’ outer surface and the positive Lap edge. The feasibility of HNTs/Lap hydrogel as a carrier for KGN was assessed by in vitro release experiments performed at pH 7.4 and in ex vivo synovial fluid at 37 °C, showing a sustained release of the drug which could be beneficial for a therapeutic treatment.

Current studies are devoted to combining the properties of HNTs with those of lamellar clays, such as hectorite by a covalent linkage between them (HNTs-Ht) [15]. Similar to HNTs, the hectorite edge, which is present in some silanol groups, can be covalently modified by grafting of organo-silane [187].

Therefore, a thiolene reaction between thiol HNTs and allyl modified hectorite is being studied. In this way, a HNTs-Ht system is synthesized which could show complementary adsorption abilities towards different biologically active species.

## Figures and Tables

**Table 1:** Physico-chemical features of halloysite nanotubes (HNT)s.

Chemical Formula	Al_2_Si_2_O_5_(OH)_4_·*n*H_2_O
Length	0.2–2 μm
Outer diameter	40–70 nm
Inner diameter	10–40 nm
Aspect ratio (L/D)	10–50
Elastic modulus (theoretical value)	140 GPa (130–340 GPa)
Mean particle size in aqueous solution	143 nm
Particle size range in aqueous solution	22.1–81.6 m^2^/g [8]
BET surface area	50–400 nm
Pore space	22.1–46.8%
Lumen space	11–395
Density	2.14–2.59 g/cm^3^
Average pore size	79.7–100.2 Å
Structural water release temperature	400–600 °C

**Table 2:** Overview of HNTs/polymer nanocomposites.

Polymer Matrix	Properties of Nanocomposites in Comparison with Neat Polymers	Ref.
Epoxy resin	Increase in thermal stability and impact strength	[24,25]
Polyvinyl alcohol	Increase in thermal stability	[26]
Polystyrene	Increase in thermal stability	[27]
Nylon 6	Increase flame retardancy	[28]
Ethylene propylene diene monomer	Increase in tensile strength, stiffness, ductility, thermal stability and flame retardancy	[29]
Epoxy/cyanate ester resin	Decrease of coefficient of thermal expansion, increase in the moduli and rubbery state	[30]
Polyaniline	Increase in the conductivity	[31]

## References

[1] Pasbakhsh P., Ismail H., Fauzi M.N.A., Bakar A.A. (2009). Influence of maleic anhydride grafted ethylene propylene diene monomer (MAH-g-EPDM) on the properties of EPDM nanocomposites reinforced by halloysite nanotubes. Polym. Test..

[2] Yuan P., Tan D., Annabi-Bergaya F. (2015). Properties and applications of halloysite nanotubes: Recent research advances and future prospects. Appl. Clay Sci..

[3] Bretti C., Cataldo S., Gianguzza A., Lando G., Lazzara G., Pettignano A., Sammartano S. (2016). Thermodynamics of Proton Binding of Halloysite Nanotubes. J. Phys. Chem. C.

[4] Veerabadran N.G., Price R.R., Lvov Y.M. (2007). Clay nanotubes for encapsulation and sustained release of drugs. Nano.

[5] Konnova S.A., Sharipova I.R., Demina T.A., Osin Y.N., Yarullina D.R., Ilinskaya O.N., Lvov Y.M., Fakhrullin R.F. (2013). Biomimetic cell-mediated three-dimensional assembly of halloysite nanotubes. Chem. Commun..

[6] Abdullayev E., Abbasov V., Tursunbayeva A., Portnov V., Ibrahimov H., Mukhtarova G., Lvov Y. (2013). Self-healing coatings based on halloysite clay polymer composites for protection of copper alloys. ACS Appl. Mater. Interfaces.

[7] Abdullayev E., Lvov Y. (2013). Halloysite clay nanotubes as a ceramic “skeleton” for functional biopolymer composites with sustained drug release. J. Mater. Chem. B.

[8] Pasbakhsh P., Churchman G.J., Keeling J.L. (2013). Characterisation of properties of various halloysites relevant to their use as nanotubes and microfibre fillers. Appl. Clay Sci..

[9] Ma W., Wu H., Higaki Y., Takahara A. (2018). Halloysite Nanotubes: Green Nanomaterial for Functional Organic-Inorganic Nanohybrids. Chem. Rec..

[10] Lisuzzo L., Cavallaro G., Lazzara G., Milioto S., Parisi F., Stetsyshyn Y. (2018). Stability of Halloysite, Imogolite, and Boron Nitride Nanotubes in Solvent Media. Appl. Sci..

[11] Vergaro V., Abdullayev E., Lvov Y.M., Zeitoun A., Cingolani R., Rinaldi R., Leporatti S. (2010). Cytocompatibility and Uptake of Halloysite Clay Nanotubes. Biomacromolecules.

[12] Yah W.O., Xu H., Soejima H., Ma W., Lvov Y., Takahara A. (2012). Biomimetic Dopamine Derivative for Selective Polymer Modification of Halloysite Nanotube Lumen. J. Am. Chem. Soc..

[13] Massaro M., Barone G., Biddeci G., Cavallaro G., Di Blasi F., Lazzara G., Nicotra G., Spinella C., Spinelli G., Riela S. (2019). Halloysite nanotubes-carbon dots hybrids multifunctional nanocarrier with positive cell target ability as a potential non-viral vector for oral gene therapy. J. Colloid Interface Sci..

[14] Fakhrullina G.I., Akhatova F.S., Lvov Y.M., Fakhrullin R.F. (2015). Toxicity of halloysite clay nanotubes in vivo: A Caenorhabditis elegans study. Environ. Sci. Nano.

[15] Massaro M., Viseras Iborra C., Cavallaro G., Colletti C.G., García-Villén F., Lazzarad G., Riela S. (2020). Advanced material based on halloysite and hectorite clay minerals covalently linked with complementary properties. Appl. Clay Sci..

[16] Price R., Gaber B.P., Lvov Y. (2001). In-vitro release characteristics of tetracycline HCl, khellin and nicotinamide adenine dineculeotide from halloysite; a cylindrical mineral. J. Microencapsul..

[17] Rudolph A.S., Stilwell G., Cliff R.O., Kahn B., Spargo B.J., Rollwagen F., Monroy R.L. (1992). Biocompatibility of lipid microcylinders: Effect on cell growth and antigen presentation in culture. Biomaterials.

[18] Levis S.R., Deasy P.B. (2002). Characterisation of halloysite for use as a microtubular drug delivery system. Int. J. Pharm..

[19] Lvov Y., Price R., Gaber B., Ichinose I. (2002). Thin film nanofabrication via layer-by-layer adsorption of tubule halloysite, spherical silica, proteins and polycations. Colloids Surf. Physicochem. Eng. Asp..

[20] Levis S.R., Deasy P.B. (2003). Use of coated microtubular halloysite for the sustained release of diltiazem hydrochloride and propranolol hydrochloride. Int. J. Pharm..

[21] Kelly H.M., Deasy P.B., Ziaka E., Claffey N. (2004). Formulation and preliminary in vivo dog studies of a novel drug delivery system for the treatment of periodontitis. Int. J. Pharm..

[22] Shchukin D.G., Sukhorukov G.B., Price R.R., Lvov Y.M. (2005). Halloysite Nanotubes as Biomimetic Nanoreactors. Small.

[23] Du M., Guo B., Jia D. (2006). Thermal stability and flame retardant effects of halloysite nanotubes on poly(propylene). Eur. Polym. J..

[24] Ye Y., Chen H., Wu J., Ye L. (2007). High impact strength epoxy nanocomposites with natural nanotubes. Polymer.

[25] Liu M., Guo B., Du M., Lei Y., Jia D. (2008). Natural inorganic nanotubes reinforced epoxy resin nanocomposites. J. Polym. Res..

[26] Liu M., Guo B., Du M., Jia D. (2007). Drying induced aggregation of halloysite nanotubes in polyvinyl alcohol/halloysite nanotubes solution and its effect on properties of composite film. Appl. Phys. A.

[27] Zhao M., Liu P. (2008). Halloysite nanotubes/polystyrene (HNTs/PS) nanocomposites via in situ bulk polymerization. J. Therm. Anal. Calorim..

[28] Marney D.C.O., Russell L.J., Wu D.Y., Nguyen T., Cramm D., Rigopoulos N., Wright N., Greaves M. (2008). The suitability of halloysite nanotubes as a fire retardant for nylon 6. Polym. Degrad. Stab..

[29] Ismail H., Pasbakhsh P., Fauzi M.N.A., Abu Bakar A. (2008). Morphological, thermal and tensile properties of halloysite nanotubes filled ethylene propylene diene monomer (EPDM) nanocomposites. Polym. Test..

[30] Liu M., Guo B., Du M., Cai X., Jia D. (2007). Properties of halloysite nanotube-epoxy resin hybrids and the interfacial reactions in the systems. Nanotechnology.

[31] Zhang L., Wang T., Liu P. (2008). Polyaniline-coated halloysite nanotubes via in-situ chemical polymerization. Appl. Surf. Sci..

[32] Liu M., Guo B., Zou Q., Du M., Jia D. (2008). Interactions between halloysite nanotubes and 2,5-bis(2-benzoxazolyl) thiophene and their effects on reinforcement of polypropylene/halloysite nanocomposites. Nanotechnology.

[33] Guo B., Chen F., Lei Y., Liu X., Wan J., Jia D. (2009). Styrene-butadiene rubber/halloysite nanotubes nanocomposites modified by sorbic acid. Appl. Surf. Sci..

[34] Du M., Guo B., Jia D. (2010). Newly emerging applications of halloysite nanotubes: A review. Polym. Int..

[35] Lei Y., Tang Z., Zhu L., Guo B., Jia D. (2011). Functional thiol ionic liquids as novel interfacial modifiers in SBR/HNTs composites. Polymer.

[36] Lin Y., Ng K.M., Chan C.-M., Sun G., Wu J. (2011). High-impact polystyrene/halloysite nanocomposites prepared by emulsion polymerization using sodium dodecyl sulfate as surfactant. J. Colloid Interface Sci..

[37] Wu S., Qiu M., Guo B., Zhang L., Lvov Y. (2017). Nanodot-Loaded Clay Nanotubes as Green and Sustained Radical Scavengers for Elastomer. ACS Sustain. Chem. Eng..

[38] Li Z., Liu L., Jiménez González A., Wang D.-Y. (2017). Bioinspired polydopamine-induced assembly of ultrafine Fe(OH)_3_ nanoparticles on halloysite toward highly efficient fire retardancy of epoxy resin via an action of interfacial catalysis. Polym. Chem..

[39] Pereira M.F.R., Soares S.F., Órfão J.J.M., Figueiredo J.L. (2003). Adsorption of dyes on activated carbons: Influence of surface chemical groups. Carbon.

[40] Parida S.K., Dash S., Patel S., Mishra B.K. (2006). Adsorption of organic molecules on silica surface. Adv. Colloid Interface Sci..

[41] Yan Y., Zhang M., Gong K., Su L., Guo Z., Mao L. (2005). Adsorption of Methylene Blue Dye onto Carbon Nanotubes:  A Route to an Electrochemically Functional Nanostructure and Its Layer-by-Layer Assembled Nanocomposite. Chem. Mater..

[42] Zhao M., Liu P. (2008). Adsorption behavior of methylene blue on halloysite nanotubes. Microporous Mesoporous Mater..

[43] Jinhua W., Xiang Z., Bing Z., Yafei Z., Rui Z., Jindun L., Rongfeng C. (2010). Rapid adsorption of Cr (VI) on modified halloysite nanotubes. Desalination.

[44] Xie Y., Qian D., Wu D., Ma X. (2011). Magnetic halloysite nanotubes/iron oxide composites for the adsorption of dyes. Chem. Eng. J..

[45] Duan J., Liu R., Chen T., Zhang B., Liu J. (2012). Halloysite nanotube-Fe3O4 composite for removal of methyl violet from aqueous solutions. Desalination.

[46] Wang R., Jiang G., Ding Y., Wang Y., Sun X., Wang X., Chen W. (2011). Photocatalytic Activity of Heterostructures Based on TiO_2_ and Halloysite Nanotubes. ACS Appl. Mater. Interfaces.

[47] Ballav N., Choi H.J., Mishra S.B., Maity A. (2014). Polypyrrole-coated halloysite nanotube clay nanocomposite: Synthesis, characterization and Cr(VI) adsorption behaviour. Appl. Clay Sci..

[48] Cavallaro G., Lazzara G., Milioto S., Palmisano G., Parisi F. (2014). Halloysite nanotube with fluorinated lumen: Non-foaming nanocontainer for storage and controlled release of oxygen in aqueous media. J. Colloid Interface Sci..

[49] Owoseni O., Nyankson E., Zhang Y., Adams S.J., He J., McPherson G.L., Bose A., Gupta R.B., John V.T. (2014). Release of Surfactant Cargo from Interfacially-Active Halloysite Clay Nanotubes for Oil Spill Remediation. Langmuir.

[50] Niu M., Yang H., Zhang X., Wang Y., Tang A. (2016). Amine-Impregnated Mesoporous Silica Nanotube as an Emerging Nanocomposite for CO_2_ Capture. ACS Appl. Mater. Interfaces.

[51] Jiang L., Huang Y., Liu T. (2015). Enhanced visible-light photocatalytic performance of electrospun carbon-doped TiO_2_/halloysite nanotube hybrid nanofibers. J. Colloid Interface Sci..

[52] Massaro M., Riela S., Cavallaro G., Colletti C.G., Milioto S., Noto R., Lazzara G. (2016). Ecocompatible Halloysite/Cucurbit[8]uril Hybrid as Efficient Nanosponge for Pollutants Removal. ChemistrySelect.

[53] Massaro M., Armetta F., Cavallaro G., Chillura Martino D.F., Gruttadauria M., Lazzara G., Riela S., d’Ischia M. (2019). Effect of halloysite nanotubes filler on polydopamine properties. J. Colloid Interface Sci..

[54] Peng H., Wu D., Wan H., Jia L., Chen G., Li J., Cao Y., Liu X., Ma R. (2019). Facile synthesis and characterization of halloysite@W_18_O_49_ nanocomposite with enhanced photocatalytic properties. Appl. Clay Sci..

[55] Massaro M., Casiello M., D’Accolti L., Lazzara G., Nacci A., Nicotra G., Noto R., Pettignano A., Spinella C., Riela S. (2020). One-pot synthesis of ZnO nanoparticles supported on halloysite nanotubes for catalytic applications. Appl. Clay Sci..

[56] Aguzzi C., Donnadio A., Quaglia G., Latterini L., Viseras C., Ambrogi V. (2019). Halloysite-Doped Zinc Oxide for Enhanced Sunscreening Performance. ACS Appl. Nano Mater..

[57] Machado G.S., de Freitas Castro K.A.D., Wypych F., Nakagaki S. (2008). Immobilization of metalloporphyrins into nanotubes of natural halloysite toward selective catalysts for oxidation reactions. J. Mol. Catal. A Chem..

[58] Liu P., Zhao M. (2009). Silver nanoparticle supported on halloysite nanotubes catalyzed reduction of 4-nitrophenol (4-NP). Appl. Surf. Sci..

[59] Zhai R., Zhang B., Wan Y., Li C., Wang J., Liu J. (2013). Chitosan–halloysite hybrid-nanotubes: Horseradish peroxidase immobilization and applications in phenol removal. Chem. Eng. J..

[60] Chao C., Zhang B., Zhai R., Xiang X., Liu J., Chen R. (2014). Natural Nanotube-Based Biomimetic Porous Microspheres for Significantly Enhanced Biomolecule Immobilization. ACS Sustain. Chem. Eng..

[61] Zhai R., Zhang B., Liu L., Xie Y., Zhang H., Liu J. (2010). Immobilization of enzyme biocatalyst on natural halloysite nanotubes. Catal. Commun..

[62] Tully J., Yendluri R., Lvov Y. (2016). Halloysite Clay Nanotubes for Enzyme Immobilization. Biomacromolecules.

[63] Wang L., Chen J., Rudolph V., Zhu Z. (2012). Nanotubules-supported Ru nanoparticles for preferential CO oxidation in H2-rich stream. Adv. Powder Technol..

[64] Wang L., Chen J., Ge L., Rudolph V., Zhu Z. (2013). Halloysite nanotube supported Ru nanocatalysts synthesized by the inclusion of preformed Ru nanoparticles for preferential oxidation of CO in H_2_-rich atmosphere. J. Phys. Chem. C.

[65] Chen S., Li J., Zhang Y., Zhang D., Zhu J. (2012). Effect of preparation method on halloysite supported cobalt catalysts for Fischer-Tropsch synthesis. J. Nat. Gas Chem..

[66] Machado G.S., Lima O.J.d., Ciuffi K.J., Wypych F., Nakagaki S. (2013). Iron(III) porphyrin supported on metahalloysite: An efficient and reusable catalyst for oxidation reactions. Catal. Sci. Technol..

[67] Hong M.C., Ahn H., Choi M.C., Lee Y., Kim J., Rhee H. (2014). Pd nanoparticles immobilized on PNIPAM–halloysite: Highly active and reusable catalyst for Suzuki–Miyaura coupling reactions in water. Appl. Organomet. Chem..

[68] Sun P., Liu G., Lv D., Dong X., Wu J., Wang D. (2015). Effective activation of halloysite nanotubes by piranha solution for amine modification via silane coupling chemistry. RSC Adv..

[69] Viseras M.T., Aguzzi C., Cerezo P., Viseras C., Valenzuela C. (2008). Equilibrium and kinetics of 5-aminosalicylic acid adsorption by halloysite. Microporous Mesoporous Mater..

[70] Viseras M.-T., Aguzzi C., Cerezo P., Cultrone G., Viseras C. (2009). Supramolecular structure of 5-aminosalycilic acid/halloysite composites. J. Microencapsul..

[71] Ahmed F.R., Shoaib M.H., Azhar M., Um S.H., Yousuf R.I., Hashmi S., Dar A. (2015). In-vitro assessment of cytotoxicity of halloysite nanotubes against HepG2, HCT116 and human peripheral blood lymphocytes. Colloids Surf. B Biointerfaces.

[72] Wang X., Gong J., Gui Z., Hu T., Xu X. (2018). Halloysite nanotubes-induced Al accumulation and oxidative damage in liver of mice after 30-day repeated oral administration. Environ. Toxicol..

[73] Rozhina E., Batasheva S., Gomzikova M., Naumenko E., Fakhrullin R. (2019). Multicellular spheroids formation: The synergistic effects of halloysite nanoclay and cationic magnetic nanoparticles. Colloids Surf. Physicochem. Eng. Asp..

[74] Vergaro V., Lvov Y.M., Leporatti S. (2012). Halloysite clay nanotubes for resveratrol delivery to cancer cells. Macromol. Biosci..

[75] Wei W., Abdullayev E., Hollister A., Mills D., Lvov Y.M. (2012). Clay nanotube/poly(methyl methacrylate) bone cement composites with sustained antibiotic release. Macromol. Mater. Eng..

[76] Massaro M., Cavallaro G., Colletti C.G., D’Azzo G., Guernelli S., Lazzara G., Pieraccini S., Riela S. (2018). Halloysite nanotubes for efficient loading, stabilization and controlled release of insulin. J. Colloid Interface Sci..

[77] Kurczewska J., Pecyna P., Ratajczak M., Gajęcka M., Schroeder G. (2017). Halloysite nanotubes as carriers of vancomycin in alginate-based wound dressing. Saudi Pharm. J..

[78] Carazo E., Borrego-Sánchez A., García-Villén F., Sánchez-Espejo R., Aguzzi C., Viseras C., Sainz-Díaz C.I., Cerezo P. (2017). Assessment of halloysite nanotubes as vehicles of isoniazid. Colloids Surf. B Biointerfaces.

[79] Tenci M., Rossi S., Aguzzi C., Carazo E., Sandri G., Bonferoni M.C., Grisoli P., Viseras C., Caramella C.M., Ferrari F. (2017). Carvacrol/clay hybrids loaded into in situ gelling films. Int. J. Pharm..

[80] Ghezzi L., Spepi A., Agnolucci M., Cristani C., Giovannetti M., Tiné M.R., Duce C. (2018). Kinetics of release and antibacterial activity of salicylic acid loaded into halloysite nanotubes. Appl. Clay Sci..

[81] Massaro M., Lazzara G., Noto R., Riela S. (2020). Halloysite nanotubes: A green resource for materials and life sciences. Rend. Lincei.

[82] Massaro M., Colletti C.G., Lazzara G., Riela S. (2018). The Use of Some Clay Minerals as Natural Resources for Drug Carrier Applications. J. Funct. Biomater..

[83] Veerabadran N.G., Mongayt D., Torchilin V., Price R.R., Lvov Y.M. (2009). Organized Shells on Clay Nanotubes for Controlled Release of Macromolecules. Macromol. Rapid Commun..

[84] Ghebaur A., Garea S.A., Iovu H. (2012). New polymer–halloysite hybrid materials—potential controlled drug release system. Int. J. Pharm..

[85] Wu H., Shi Y., Huang C., Zhang Y., Wu J., Shen H., Jia N. (2014). Multifunctional nanocarrier based on clay nanotubes for efficient intracellular siRNA delivery and gene silencing. J. Biomater. Appl..

[86] Fan L., Li B., Wang Q., Wang A., Zhang J. (2014). Superhydrophobic Gated Polyorganosilanes/Halloysite Nanocontainers for Sustained Drug Release. Adv. Mater. Interface.

[87] Wei W., Minullina R., Abdullayev E., Fakhrullin R., Mills D., Lvov Y. (2014). Enhanced efficiency of antiseptics with sustained release from clay nanotubes. RSC Adv..

[88] Dzamukova M.R., Naumenko E.A., Lvov Y.M., Fakhrullin R.F. (2015). Enzyme-activated intracellular drug delivery with tubule clay nanoformulation. Sci. Rep..

[89] Arcudi F., Cavallaro G., Lazzara G., Massaro M., Milioto S., Noto R., Riela S. (2014). Selective Functionalization of Halloysite Cavity by Click Reaction: Structured Filler for Enhancing Mechanical Properties of Bionanocomposite Films. J. Phys. Chem. C.

[90] Biddeci G., Cavallaro G., Di Blasi F., Lazzara G., Massaro M., Milioto S., Parisi F., Riela S., Spinelli G. (2016). Halloysite nanotubes loaded with peppermint essential oil as filler for functional biopolymer film. Carbohydr. Polym..

[91] Makaremi M., Pasbakhsh P., Cavallaro G., Lazzara G., Aw Y.K., Lee S.M., Milioto S. (2017). Effect of Morphology and Size of Halloysite Nanotubes on Functional Pectin Bionanocomposites for Food Packaging Applications. ACS Appl. Mater. Interfaces.

[92] Krepker M., Shemesh R., Danin Poleg Y., Kashi Y., Vaxman A., Segal E. (2017). Active food packaging films with synergistic antimicrobial activity. Food Control.

[93] De Silva R.T., Pasbakhsh P., Lee S.M., Kit A.Y. (2015). ZnO deposited/encapsulated halloysite–poly (lactic acid) (PLA) nanocomposites for high performance packaging films with improved mechanical and antimicrobial properties. Appl. Clay Sci..

[94] Yendluri R., Otto D.P., De Villiers M.M., Vinokurov V., Lvov Y.M. (2017). Application of halloysite clay nanotubes as a pharmaceutical excipient. Int. J. Pharm..

[95] Sandri G., Aguzzi C., Rossi S., Bonferoni M.C., Bruni G., Boselli C., Cornaglia A.I., Riva F., Viseras C., Caramella C. (2017). Halloysite and chitosan oligosaccharide nanocomposite for wound healing. Acta Biomater..

[96] Dramou P., Fizir M., Taleb A., Itatahine A., Dahiru N.S., Mehdi Y.A., Wei L., Zhang J., He H. (2018). Folic acid-conjugated chitosan oligosaccharide-magnetic halloysite nanotubes as a delivery system for camptothecin. Carbohydr. Polym..

[97] Li L.-Y., Zhou Y.-M., Gao R.-Y., Liu X.-C., Du H.-H., Zhang J.-L., Ai X.-C., Zhang J.-P., Fu L.-M., Skibsted L.H. (2019). Naturally occurring nanotube with surface modification as biocompatible, target-specific nanocarrier for cancer phototherapy. Biomaterials.

[98] Cheng C., Gao Y., Song W., Zhao Q., Zhang H., Zhang H. (2020). Halloysite nanotube-based H_2_O_2_-responsive drug delivery system with a turn on effect on fluorescence for real-time monitoring. Chem. Eng. J..

[99] Akrami-Hasan-Kohal M., Ghorbani M., Mahmoodzadeh F., Nikzad B. (2020). Development of reinforced aldehyde-modified kappa-carrageenan/gelatin film by incorporation of halloysite nanotubes for biomedical applications. Int. J. Biol. Macromol..

[100] Rozhina E., Ishmukhametov I., Batasheva S., Akhatova F., Fakhrullin R. (2019). Nanoarchitectonics meets cell surface engineering: Shape recognition of human cells by halloysite-doped silica cell imprints. Beilstein J. Nanotechnol..

[101] Yuan P., Southon P.D., Liu Z., Green M.E.R., Hook J.M., Antill S.J., Kepert C.J. (2008). Functionalization of Halloysite Clay Nanotubes by Grafting with γ-Aminopropyltriethoxysilane. J. Phys. Chem. C.

[102] Fidecka K., Giacoboni J., Picconi P., Vago R., Licandro E. (2020). Quantification of amino groups on halloysite surfaces using the Fmoc-method. RSC Adv..

[103] Tan D., Yuan P., Annabi-Bergaya F., Yu H., Liu D., Liu H., He H. (2013). Natural halloysite nanotubes as mesoporous carriers for the loading of ibuprofen. Microporous Mesoporous Mater..

[104] Tan D., Yuan P., Annabi-Bergaya F., Liu D., Wang L., Liu H., He H. (2014). Loading and in vitro release of ibuprofen in tubular halloysite. Appl. Clay Sci..

[105] Lun H., Ouyang J., Yang H. (2014). Natural halloysite nanotubes modified as an aspirin carrier. RSC Adv..

[106] Shi Y.-F., Tian Z., Zhang Y., Shen H.-B., Jia N.-Q. (2011). Functionalized halloysite nanotube-based carrier for intracellular delivery of antisense oligonucleotides. Nanoscale Res. Lett..

[107] Liu Y., Jiang X., Li B., Zhang X., Liu T., Yan X., Ding J., Cai Q., Zhang J. (2014). Halloysite nanotubes@reduced graphene oxide composite for removal of dyes from water and as supercapacitors. J. Mater. Chem. A.

[108] Del Buffa S., Bonini M., Ridi F., Severi M., Losi P., Volpi S., Al Kayal T., Soldani G., Baglioni P. (2015). Design and characterization of a composite material based on Sr(II)-loaded clay nanotubes included within a biopolymer matrix. J. Colloid Interface Sci..

[109] Zeng G., He Y., Zhan Y., Zhang L., Pan Y., Zhang C., Yu Z. (2016). Novel polyvinylidene fluoride nanofiltration membrane blended with functionalized halloysite nanotubes for dye and heavy metal ions removal. J. Hazard. Mater..

[110] Kumar-Krishnan S., Hernandez-Rangel A., Pal U., Ceballos-Sanchez O., Flores-Ruiz F.J., Prokhorov E., Arias de Fuentes O., Esparza R., Meyyappan M. (2016). Surface functionalized halloysite nanotubes decorated with silver nanoparticles for enzyme immobilization and biosensing. J. Mater. Chem. B.

[111] Cataldo S., Lazzara G., Massaro M., Muratore N., Pettignano A., Riela S. (2018). Functionalized halloysite nanotubes for enhanced removal of lead(II) ions from aqueous solutions. Appl. Clay Sci..

[112] Cavallaro G., Lazzara G., Massaro M., Milioto S., Noto R., Parisi F., Riela S. (2015). Biocompatible poly(N-isopropylacrylamide)-halloysite nanotubes for thermoresponsive curcumin release. J. Phys. Chem. C.

[113] Massaro M., Schembri V., Campisciano V., Cavallaro G., Lazzara G., Milioto S., Noto R., Parisi F., Riela S. (2016). Design of PNIPAAM covalently grafted on halloysite nanotubes as a support for metal-based catalysts. RSC Adv..

[114] Kurczewska J., Cegłowski M., Messyasz B., Schroeder G. (2018). Dendrimer-functionalized halloysite nanotubes for effective drug delivery. Appl. Clay Sci..

[115] Hemmatpour H., Haddadi-Asl V., Roghani-Mamaqani H. (2015). Synthesis of pH-sensitive poly (N,N-dimethylaminoethyl methacrylate)-grafted halloysite nanotubes for adsorption and controlled release of DPH and DS drugs. Polymer.

[116] Eskandarloo H., Arshadi M., Abbaspourrad A. (2018). Magnetic Dendritic Halloysite Nanotube for Highly Selective Recovery of Heparin Digested from Porcine Intestinal Mucosa. ACS Sustain. Chem. Eng..

[117] Eskandarloo H., Arshadi M., Enayati M., Abbaspourrad A. (2018). Highly Efficient Recovery of Heparin Using a Green and Low-Cost Quaternary Ammonium Functionalized Halloysite Nanotube. ACS Sustain. Chem. Eng..

[118] Long Z., Zhang J., Shen Y., Zhou C., Liu M. (2017). Polyethyleneimine grafted short halloysite nanotubes for gene delivery. Mater. Sci. Eng. C.

[119] Long Z., Wu Y.-P., Gao H.-Y., Li Y.-F., He R.-R., Liu M. (2018). Functionalization of Halloysite Nanotubes via Grafting of Dendrimer for Efficient Intracellular Delivery of siRNA. Bioconjugate Chem..

[120] Kadam A.A., Jang J., Lee D.S. (2017). Supermagnetically Tuned Halloysite Nanotubes Functionalized with Aminosilane for Covalent Laccase Immobilization. ACS Appl. Mater. Interfaces.

[121] Sillu D., Agnihotri S. (2020). Cellulase Immobilization onto Magnetic Halloysite Nanotubes: Enhanced Enzyme Activity and Stability with High Cellulose Saccharification. ACS Sustain. Chem. Eng..

[122] Massaro M., Riela S., Guernelli S., Parisi F., Lazzara G., Baschieri A., Valgimigli L., Amorati R. (2016). A synergic nanoantioxidant based on covalently modified halloysite-trolox nanotubes with intra-lumen loaded quercetin. J. Mater. Chem. B.

[123] Rizzo C., Arrigo R., D’Anna F., Di Blasi F., Dintcheva N.T., Lazzara G., Parisi F., Riela S., Spinelli G., Massaro M. (2017). Hybrid supramolecular gels of Fmoc-F/halloysite nanotubes: Systems for sustained release of camptothecin. J. Mater. Chem. B.

[124] Li X., Chen J., Liu H., Deng Z., Li J., Ren T., Huang L., Chen W., Yang Y., Zhong S. (2019). β-Cyclodextrin coated and folic acid conjugated magnetic halloysite nanotubes for targeting and isolating of cancer cells. Colloids Surf. B. Biointerfaces.

[125] Yu T., Swientoniewski L.T., Omarova M., Li M.-C., Negulescu I.I., Jiang N., Darvish O.A., Panchal A., Blake D.A., Wu Q. (2019). Investigation of Amphiphilic Polypeptoid-Functionalized Halloysite Nanotubes as Emulsion Stabilizer for Oil Spill Remediation. ACS Appl. Mater. Interf..

[126] Su Z., Zhang H., Gao Y., Huo L., Wu Y., Ba X. (2020). Coumarin-anchored halloysite nanotubes for highly selective detection and removal of Zn(II). Chem. Eng. J..

[127] Taroni T., Cauteruccio S., Vago R., Franchi S., Barbero N., Licandro E., Ardizzone S., Meroni D. (2020). Thiahelicene-grafted halloysite nanotubes: Characterization, biological studies and pH triggered release. Appl. Surf. Sci..

[128] Joo Y., Jeon Y., Lee S.U., Sim J.H., Ryu J., Lee S., Lee H., Sohn D. (2012). Aggregation and Stabilization of Carboxylic Acid Functionalized Halloysite Nanotubes (HNT-COOH). J. Phys. Chem. C.

[129] Pan J., Yao H., Xu L., Ou H., Huo P., Li X., Yan Y. (2011). Selective Recognition of 2,4,6-Trichlorophenol by Molecularly Imprinted Polymers Based on Magnetic Halloysite Nanotubes Composites. J. Phys. Chem. C.

[130] Liu M., Chang Y., Yang J., You Y., He R., Chen T., Zhou C. (2016). Functionalized halloysite nanotube by chitosan grafting for drug delivery of curcumin to achieve enhanced anticancer efficacy. J. Mater. Chem. B.

[131] Yang J., Wu Y., Shen Y., Zhou C., Li Y.-F., He R.-R., Liu M. (2016). Enhanced Therapeutic Efficacy of Doxorubicin for Breast Cancer Using Chitosan Oligosaccharide-Modified Halloysite Nanotubes. ACS Appl. Mater. Interfaces.

[132] Yamina A.M., Fizir M., Itatahine A., He H., Dramou P. (2018). Preparation of multifunctional PEG-graft-Halloysite Nanotubes for Controlled Drug Release, Tumor Cell Targeting, and Bio-imaging. Colloids Surf. B Biointerfaces.

[133] Massaro M., Colletti C.G., Guernelli S., Lazzara G., Liu M., Nicotra G., Noto R., Parisi F., Pibiri I., Spinella C. (2018). Photoluminescent hybrid nanomaterials from modified halloysite nanotubes. J. Mater. Chem. C.

[134] Zhang Y., Pan J., Gan M., Ou H., Yan Y., Shi W., Yu L. (2014). Acid–chromic chloride functionalized natural clay-particles for enhanced conversion of one-pot cellulose to 5-hydroxymethylfurfural in ionic liquids. RSC Adv..

[135] Massaro M., Riela S., Cavallaro G., Gruttadauria M., Milioto S., Noto R., Lazzara G. (2014). Eco-friendly functionalization of natural halloysite clay nanotube with ionic liquids by microwave irradiation for Suzuki coupling reaction. J. Organomet. Chem..

[136] Massaro M., Riela S., Lazzara G., Gruttadauria M., Milioto S., Noto R. (2014). Green conditions for the Suzuki reaction using microwave irradiation and a new HNT-supported ionic liquid-like phase (HNT-SILLP) catalyst. Appl. Organomet. Chem..

[137] Bellani L., Giorgetti L., Riela S., Lazzara G., Scialabba A., Massaro M. (2016). Ecotoxicity of halloysite nanotube–supported palladium nanoparticles in *Raphanus sativus* L.. Environ. Toxicol. Chem..

[138] Massaro M., Colletti C.G., Buscemi G., Cataldo S., Guernelli S., Lazzara G., Liotta L.F., Parisi F., Pettignano A., Riela S. (2018). Palladium nanoparticles immobilized on halloysite nanotubes covered by a multilayer network for catalytic applications. New J. Chem..

[139] Massaro M., Riela S., Lo Meo P., Noto R., Cavallaro G., Milioto S., Lazzara G. (2014). Functionalized halloysite multivalent glycocluster as a new drug delivery system. J. Mater. Chem. B.

[140] Massaro M., Riela S., Baiamonte C., Blanco J.L.J., Giordano C., Lo Meo P., Milioto S., Noto R., Parisi F., Pizzolanti G. (2016). Dual drug-loaded halloysite hybrid-based glycocluster for sustained release of hydrophobic molecules. RSC Adv..

[141] Massaro M., Piana S., Colletti C.G., Noto R., Riela S., Baiamonte C., Giordano C., Pizzolanti G., Cavallaro G., Milioto S. (2015). Multicavity halloysite-amphiphilic cyclodextrin hybrids for co-delivery of natural drugs into thyroid cancer cells. J. Mater. Chem. B.

[142] Massaro M., Amorati R., Cavallaro G., Guernelli S., Lazzara G., Milioto S., Noto R., Poma P., Riela S. (2016). Direct chemical grafted curcumin on halloysite nanotubes as dual-responsive prodrug for pharmacological applications. Colloids Surf. B. Biointerfaces.

[143] Massaro M., Colletti C.G., Lazzara G., Guernelli S., Noto R., Riela S. (2017). Synthesis and Characterization of Halloysite-Cyclodextrin Nanosponges for Enhanced Dyes Adsorption. ACS Sustain. Chem. Eng..

[144] Massaro M., Campofelice A., Colletti C.G., Lazzara G., Noto R., Riela S. (2018). Functionalized halloysite nanotubes: Efficient carrier systems for antifungine drugs. Appl. Clay Sci..

[145] Massaro M., Riela S. (2018). Organo-clay nanomaterials based on halloysite and cyclodextrin as carriers for polyphenolic compounds. J. Funct. Biomater..

[146] Massaro M., Colletti C.G., Fiore B., La Parola V., Lazzara G., Guernelli S., Zaccheroni N., Riela S. (2019). Gold nanoparticles stabilized by modified halloysite nanotubes for catalytic applications. Appl. Organomet. Chem..

[147] Cheng D., Dai X., Chen L., Cui Y., Qiang C., Sun Q., Dai J. (2020). Thiol–Yne Click Synthesis of Polyamide–Amine Dendritic Magnetic Halloysite Nanotubes for the Efficient Removal of Pb(II). ACS Sustain. Chem. Eng..

[148] Pasbakhsh P., Ismail H., Fauzi M.N.A., Bakar A.A. (2010). EPDM/modified halloysite nanocomposites. Appl. Clay Sci..

[149] Murariu M., Dechief A.L., Paint Y., Peeterbroeck S., Bonnaud L., Dubois P. (2012). Polylactide (PLA)-Halloysite Nanocomposites: Production, Morphology and Key-Properties. J. Polym. Environ..

[150] Zhong S., Zhou C., Zhang X., Zhou H., Li H., Zhu X., Wang Y. (2014). A novel molecularly imprinted material based on magnetic halloysite nanotubes for rapid enrichment of 2,4-dichlorophenoxyacetic acid in water. J. Hazard. Mater..

[151] Dai J., Wei X., Cao Z., Zhou Z., Yu P., Pan J., Zou T., Li C., Yan Y. (2014). Highly-controllable imprinted polymer nanoshell at the surface of magnetic halloysite nanotubes for selective recognition and rapid adsorption of tetracycline. RSC Adv..

[152] Fu Y., Zhao D., Yao P., Wang W., Zhang L., Lvov Y. (2015). Highly Aging-Resistant Elastomers Doped with Antioxidant-Loaded Clay Nanotubes. ACS Appl. Mater. Interfaces.

[153] Riela S., Massaro M., Colletti C.G., Bommarito A., Giordano C., Milioto S., Noto R., Poma P., Lazzara G. (2014). Development and characterization of co-loaded curcumin/triazole-halloysite systems and evaluation of their potential anticancer activity. Int. J. Pharm..

[154] Massaro M., Colletti C.G., Noto R., Riela S., Poma P., Guernelli S., Parisi F., Milioto S., Lazzara G. (2015). Pharmaceutical properties of supramolecular assembly of co-loaded cardanol/triazole-halloysite systems. Int. J. Pharm..

[155] Massaro M., Riela S., Cavallaro G., Colletti C.G., Milioto S., Noto R., Parisi F., Lazzara G. (2015). Palladium supported on Halloysite-triazolium salts as catalyst for ligand free Suzuki cross-coupling in water under microwave irradiation. J. Mol. Catal. A Chem..

[156] Massaro M., Poma P., Colletti C.G., Barattucci A., Bonaccorsi P.M., Lazzara G., Nicotra G., Parisi F., Salerno T.M.G., Spinella C. (2020). Chemical and biological evaluation of cross-linked halloysite-curcumin derivatives. Appl. Clay Sci..

[157] Barrientos-Ramírez S., Oca-Ramírez G.M.d., Ramos-Fernández E.V., Sepúlveda-Escribano A., Pastor-Blas M.M., González-Montiel A. (2011). Surface modification of natural halloysite clay nanotubes with aminosilanes. Application as catalyst supports in the atom transfer radical polymerization of methyl methacrylate. Appl. Catal. A.

[158] Luo P., Zhang J.-S., Zhang B., Wang J.-H., Zhao Y.-F., Liu J.-D. (2011). Preparation and Characterization of Silane Coupling Agent Modified Halloysite for Cr(VI) Removal. Ind. Eng. Chem. Res..

[159] Zhang J., Zhang Y., Chen Y., Du L., Zhang B., Zhang H., Liu J., Wang K. (2012). Preparation and Characterization of Novel Polyethersulfone Hybrid Ultrafiltration Membranes Bending with Modified Halloysite Nanotubes Loaded with Silver Nanoparticles. Ind. Eng. Chem. Res..

[160] Jiang J., Zhang Y., Yan L., Jiang P. (2012). Epoxidation of soybean oil catalyzed by peroxo phosphotungstic acid supported on modified halloysite nanotubes. Appl. Surf. Sci..

[161] Bahri-Laleh N., Sadjadi S., Poater A. (2018). Pd immobilized on dendrimer decorated halloysite clay: Computational and experimental study on the effect of dendrimer generation, Pd valance and incorporation of terminal functionality on the catalytic activity. J. Colloid Interface Sci..

[162] Ding X., Wang H., Chen W., Liu J., Zhang Y. (2014). Preparation and antibacterial activity of copper nanoparticle/halloysite nanotube nanocomposites via reverse atom transfer radical polymerization. RSC Adv..

[163] Hou Y., Jiang J., Li K., Zhang Y., Liu J. (2014). Grafting Amphiphilic Brushes onto Halloysite Nanotubes via a Living RAFT Polymerization and Their Pickering Emulsification Behavior. J. Phys. Chem. B.

[164] Ataee-Kachouei T., Nasr-Esfahani M., Mohammadpoor-Baltork I., Mirkhani V., Moghadam M., Tangestaninejad S., Notash B. (2020). Ce(IV) immobilized on halloysite nanotube–functionalized dendrimer (Ce(IV)–G2): A novel and efficient dendritic catalyst for the synthesis of pyrido[3,2-c]coumarin derivatives. Appl. Organomet. Chem..

[165] Li H., Zhu X., Zhou H., Zhong S. (2015). Functionalization of halloysite nanotubes by enlargement and hydrophobicity for sustained release of analgesic. Colloids Surf. Physicochem. Eng. Asp..

[166] Feng K., Hung G.-Y., Liu J., Li M., Zhou C., Liu M. (2018). Fabrication of high performance superhydrophobic coatings by spray-coating of polysiloxane modified halloysite nanotubes. Chem. Eng. J..

[167] Wu F., Pickett K., Panchal A., Liu M., Lvov Y. (2019). Superhydrophobic Polyurethane Foam Coated with Polysiloxane-Modified Clay Nanotubes for Efficient and Recyclable Oil Absorption. ACS Appl. Mater. Interfaces.

[168] Zhu K., Duan Y., Wang F., Gao P., Jia H., Ma C., Wang C. (2017). Silane-modified halloysite/Fe3O4 nanocomposites: Simultaneous removal of Cr(VI) and Sb(V) and positive effects of Cr(VI) on Sb(V) adsorption. Chem. Eng. J..

[169] Zhao S., Yuan Y., Yu Q., Niu B., Liao J., Guo Z., Wang N. (2019). A Dual-Surface Amidoximated Halloysite Nanotube for High-Efficiency Economical Uranium Extraction from Seawater. Angew. Chem. Int. Ed..

[170] Yah W.O., Takahara A., Lvov Y.M. (2012). Selective Modification of Halloysite Lumen with Octadecylphosphonic Acid: New Inorganic Tubular Micelle. J. Am. Chem. Soc..

[171] Jing H., Higaki Y., Ma W., Wu H., Yah W.O., Otsuka H., Lvov Y.M., Takahara A. (2013). Internally Modified Halloysite Nanotubes as Inorganic Nanocontainers for a Flame Retardant. Chem. Lett..

[172] Zhang H., Ren T., Ji Y., Han L., Wu Y., Song H., Bai L., Ba X. (2015). Selective Modification of Halloysite Nanotubes with 1-Pyrenylboronic Acid: A Novel Fluorescence Probe with Highly Selective and Sensitive Response to Hyperoxide. ACS Appl. Mater. Interfaces.

[173] Liu F., Bai L., Zhang H., Song H., Hu L., Wu Y., Ba X. (2017). Smart H_2_O_2_-Responsive Drug Delivery System Made by Halloysite Nanotubes and Carbohydrate Polymers. ACS Appl. Mater. Interfaces.

[174] Dedzo K.G., Ngnie G., Detellier C. (2016). PdNP Decoration of Halloysite Lumen via Selective Grafting of Ionic Liquid onto the Aluminol Surfaces and Catalytic Application. ACS Appl. Mater. Interfaces.

[175] Joussein E., Petit S., Delvaux B. (2007). Behavior of halloysite clay under formamide treatment. Appl. Clay Sci..

[176] Nicolini K.P., Fukamachi C.R.B., Wypych F., Mangrich A.S. (2009). Dehydrated halloysite intercalated mechanochemically with urea: Thermal behavior and structural aspects. J. Colloid Interface Sci..

[177] Mellouk S., Belhakem A., Marouf-Khelifa K., Schott J., Khelifa A. (2011). Cu(II) adsorption by halloysites intercalated with sodium acetate. J. Colloid Interface Sci..

[178] Tang Y., Deng S., Ye L., Yang C., Yuan Q., Zhang J., Zhao C. (2011). Effects of unfolded and intercalated halloysites on mechanical properties of halloysite–epoxy nanocomposites. Compos. Part A.

[179] White R.D., Bavykin D.V., Walsh F.C. (2012). The stability of halloysite nanotubes in acidic and alkaline aqueous suspensions. Nanotechnology.

[180] Zhang A.-B., Pan L., Zhang H.-Y., Liu S.-T., Ye Y., Xia M.-S., Chen X.-G. (2012). Effects of acid treatment on the physico-chemical and pore characteristics of halloysite. Colloids Surf. Physicochem. Eng. Asp..

[181] Abdullayev E., Joshi A., Wei W., Zhao Y., Lvov Y. (2012). Enlargement of Halloysite Clay Nanotube Lumen by Selective Etching of Aluminum Oxide. ACS Nano.

[182] Szczepanik B., Słomkiewicz P., Garnuszek M., Czech K. (2014). Adsorption of chloroanilines from aqueous solutions on the modified halloysite. Appl. Clay Sci..

[183] Wang Q., Zhang J., Zheng Y., Wang A. (2014). Adsorption and release of ofloxacin from acid- and heat-treated halloysite. Colloids Surf. B Biointerfaces.

[184] Garcia-Garcia D., Ferri J.M., Ripoll L., Hidalgo M., Lopez-Martinez J., Balart R. (2017). Characterization of selectively etched halloysite nanotubes by acid treatment. Appl. Surf. Sci..

[185] Lisuzzo L., Wicklein B., Lo Dico G., Lazzara G., del Real G., Aranda P., Ruiz-Hitzky E. (2020). Functional biohybrid materials based on halloysite, sepiolite and cellulose nanofibers for health applications. Dalton Trans..

[186] Massaro M., Buscemi G., Arista L., Biddeci G., Cavallaro G., D’Anna F., Di Blasi F., Ferrante A., Lazzara G., Rizzo C. (2019). Multifunctional Carrier Based on Halloysite/Laponite Hybrid Hydrogel for Kartogenin Delivery. ACS Med. Chem. Lett..

[187] Colletti C.G., Massaro M., Lazzara G., Cavallaro G., Milioto S., Pibiri I., Noto R., Riela S. (2020). Synthesis, characterization and study of covalently modified triazole laponite^®^ edges. Appl. Clay Sci..

